# TATTOO-seq delineates spatial and cell type–specific regulatory programs in the developing limb

**DOI:** 10.1126/sciadv.add0695

**Published:** 2022-12-14

**Authors:** Sébastien Bastide, Elad Chomsky, Baptiste Saudemont, Yann Loe-Mie, Sandrine Schmutz, Sophie Novault, Heather Marlow, Amos Tanay, François Spitz

**Affiliations:** ^1^ (Epi)genomics of Animal Development, Department of Developmental and Stem Cell Biology, Institut Pasteur, Paris, France.; ^2^ École Doctorale “Complexité du Vivant”, Sorbonne Université, 75005 Paris, France.; ^3^ Department of Human Genetics, The University of Chicago, Chicago, IL, USA.; ^4^ Department of Computer Science and Applied Mathematics, Weizmann Institute, Rehovot, Israel.; ^5^ Department of Biological Regulation, Weizmann Institute, Rehovot, Israel.; ^6^ Hub de Bioinformatique et Biostatistique, Département Biologie Computationnelle, Institut Pasteur, Paris, France.; ^7^ Cytometry and Biomarkers, Center for Technological Resources and Research, Institut Pasteur, Paris, France.; ^8^ Department of Organismal Biology and Anatomy, The University of Chicago, Chicago, IL, USA.

## Abstract

The coordinated differentiation of progenitor cells into specialized cell types and their spatial organization into distinct domains is central to embryogenesis. Here, we developed and applied an unbiased spatially resolved single-cell transcriptomics method to identify the genetic programs underlying the emergence of specialized cell types during mouse limb development and their spatial integration. We identify multiple transcription factors whose expression patterns are predominantly associated with cell type specification or spatial position, suggesting two parallel yet highly interconnected regulatory systems. We demonstrate that the embryonic limb undergoes a complex multiscale reorganization upon perturbation of one of its spatial organizing centers, including the loss of specific cell populations, alterations of preexisting cell states’ molecular identities, and changes in their relative spatial distribution. Our study shows how multidimensional single-cell, spatially resolved molecular atlases can allow the deconvolution of spatial identity and cell fate and reveal the interconnected genetic networks that regulate organogenesis and its reorganization upon genetic alterations.

## Teaser

Spatially resolved scRNA-seq deconvolutes positional and cell type-specific components of cell identity in the mouse limb bud.

## INTRODUCTION

During embryonic development, cells acquire different identities according to a specific patterning blueprint, which ensures that organs and other structures are correctly positioned along the body plan (*1*). Impaired establishment of spatial cues or the inability of cells to correctly acquire and interpret positional information can lead to developmental abnormalities (*2*, *3*), and modulation of patterning centers’ activity has been suggested to underlie evolutionary morphological diversification (*4*–*7*). The recent development of single-cell transcriptomics has allowed the characterization of cell state diversity within a tissue and to identify the associated gene signatures (*8*). However, spatial position is usually lost in the process, which is problematic in the context of embryonic patterning. Several methods have been developed to preserve or retrieve spatial information in single-cell data, each with specific strengths, limitations, and trade-offs. Retrospective mapping of single-cell clusters using spatial landmark genes (*9*, *10*) relies on arbitrary binary thresholds and may become impractical when experimental conditions affect landmark genes. Highly multiplexed single-molecule RNA–fluorescence in situ hybridization (FISH) (multiplexed error-robust–FISH and sequential FISH) (*11*, *12*) and in situ sequencing methods (fluorescent in situ sequencing and spatially-resolved transcript amplicon readout mapping) (*13*, *14*) provide direct quantitative spatial measurements, but their detection power remains limited to a couple thousands of genes simultaneously. Spatially resolved mRNA captured via barcoded beads (Slide-seq and Stereo-seq) (*15*, *16*) offers high spatial resolution for tissue sections but yields sparse datasets that impede lowly expressed gene detection and does not currently provide single-cell resolution. Furthermore, both approaches remain technically challenging, requiring specialized equipment and reagents, and often inconvenient for large specimens. Here, we present a simple alternative approach, TATTOO-seq, allowing the characterization of thousands of cells by single-cell RNA sequencing (scRNA-seq), while independently recording their spatial position of origin and transcriptome.

As a proof of principle, we applied TATTOO-seq to characterize the genetic programs coordinating spatial patterning and cell identity in the developing mouse limb, a classic model of patterning and differentiation. In the limb, secreted fibroblast growth factors (FGFs) from the distal apical ectodermal ridge (AER) and sonic hedgehog (SHH) from the posterior zone of polarizing activity (ZPA) define a coordinate system along the proximal-distal (PD) and anterior-posterior (AP) axes, respectively (*7*, *17*). Within this patterned field, mesenchymal progenitor cells, migrating myoblasts, and neurons will differentiate and organize into cartilage and bone, muscles, tendons, ligaments, connective tissues, nerves, and blood vessels (*18*–*20*). Undifferentiated mesenchymal precursors that populate the limb bud expand at different rates and adopt different fates depending on their location. Notably, chondrogenic condensations form in response to these positional cues and build a cartilage anlagen that will later on be ossified. Others make up the fibrous connective tissue that connects the musculoskeletal system: tendons and ligaments. Spatial information thus directly affects the number, position, shape, and length of the skeletal elements, as well as the attachment of muscle and connective tissues that make up a functional limb. Accordingly, the extensive diversity of limb morphologies observed in tetrapods often seems to result from changes to early limb patterning mechanisms or their interpretation by chondrogenic progenitors (*4*, *5*, *7*, *21*). Similarly, alterations in the genes regulating the formation and activity of the limb patterning centers underlie many of the limb malformations found in human patients [e.g., (*22*–*25*)]. Although decades of genetic studies have identified the signaling pathways (*26*, *27*) and transcription factors (TFs) (*28*–*31*) controlling limb patterning and the differentiation of its cell types, we still lack a comprehensive understanding on how individual cells define and adapt their gene regulatory programs according to their position. Furthermore, while limb patterning has been extensively studied in the context of the formation of the skeletal elements, the influence of patterning signals on the organization and specialization of the other types of connective tissues are far less understood.

## RESULTS

### An unbiased strategy for spatially resolved scRNA-seq

**Fig. 1.**
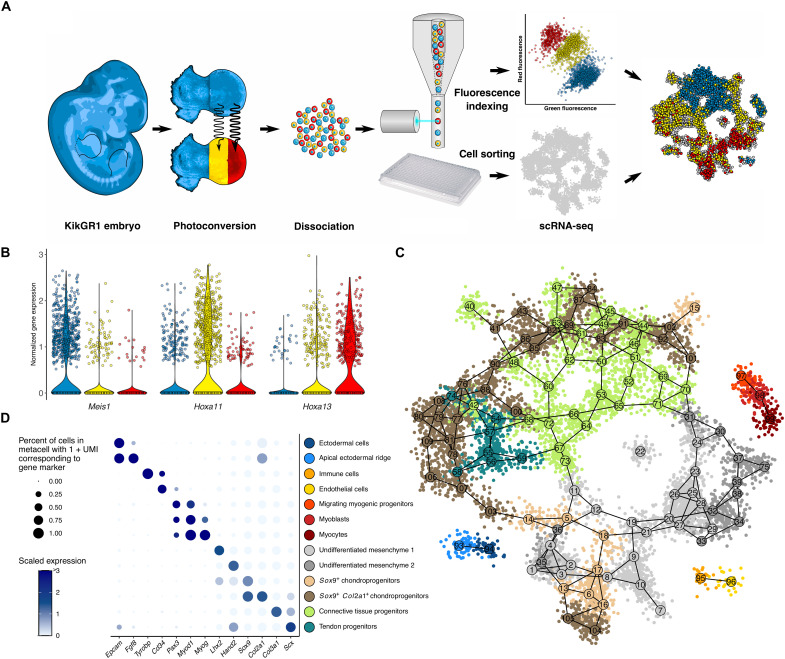
TATTOO-seq simultaneously records the position and measures the transcriptome of single cells in the mouse limb bud. (**A**) Overview of TATTOO-seq. (**B**) Expression of PD markers *Meis1*, *Hoxa11*, and *Hoxa13* (*78*) for each color along the PD axis. (**C**) Two-dimensional projection of the metacell graph. (**D**) Marker gene expression in each annotated cell type.

TATTOO-seq is based on an optical registration system (Fig. 1A) using the constitutively expressed photoconvertible protein Kikume Green-Red (KikGR1) (*32*). The modulation of the degree of photoconversion of cellular KikGR1 proteins produces distinct levels of red-to-green fluorescence ratios, which are used as a positional index in a flexible, user-defined, spatial coordinate system. This index can be recorded as cells are fluorescence-activated cell sorting (FACS)–sorted into barcoded 384-well plates and processed by massively parallel scRNA-seq (MARS-seq) (*33*). This strategy enables the independent determination of single-cell positions and transcriptomes, with improved flexibility over simple photoactivation [as used for NICHE-seq (*34*)].

We used TATTOO-seq to study patterning and differentiation in embryonic day 10.5 (E10.5) to E11.5 mouse forelimb buds. Under our experimental conditions, photoconversion was homogeneous throughout the limb thickness and did not affect cell viability or gene expression (fig. S1). To characterize the developing limb bud transcriptional landscape, we sorted ~10,000 live cells from E11.5 forelimbs (45 to 51 somites; embryos, *n* = 4; limbs, *n* = 7) and performed MARS-seq (*33*). A total of 8750 cells were kept after quality control filtering [unique molecular identifier (UMI) > 2000] (fig. S2A). To limit batch effects, we used different photoconversion patterns on the two forelimbs of each embryo. We first examined the efficiency of the KikGR1-encoded optical registration method to correctly identify the position of origin of cells. We considered the expression of the positional markers *Meis1* (proximal), *Hoxa11* (medial), and *Hoxa13* (distal) (*17*) in samples photoconverted along the PD axis (Fig. 1B). The expression of these markers showed strong concurrence with the expected indexed color, validating that TATTOO-seq accurately retains positional information.

### A molecular atlas of the developing limb bud

We used MetaCell (*35*) to identify transcriptionally homogeneous groups of cells and reconstruct the developing limb bud’s transcriptional manifold. We identified 109 metacells (Fig. 1C). As a control for batch- or stage-specific biases, we analyzed the contribution of each embryo to the different metacells (fig. S2, B and C). The vast majority of the metacells comprised cells from all four embryos, indicating that the corresponding cell states are present in the limb throughout the period analyzed (45 to 51 somites). We, however, noticed that metacells 5, 17 to 19, 21, and 27 showed a biased contribution from embryo #2 (45 somites), but not from embryo #5 (45 somites as well), a bias that could be explained by the specific sampling of medial and distal cell states in sample #2 or a batch effect. For example, the transcriptome of metacell 27 (mostly from embryo #2) is very similar to that of metacell 26 (which is depleted from cells from embryo #2). This suggests that metacells 26 and 27 represent the same cell state, up to a batch effect. We then assigned each metacell to the different cell populations present in the limb bud using known cell type–specific markers (Fig. 1D and fig. S3). The average expression in each metacell (as a ratio to the median expression in all metacells) is provided in table S1. Mesenchymal cell states constituted the bulk of the data and formed a continuum of related cell states. Multiple metacells represented the chondrogenic lineage, comprising *Sox9*^+^-condensing chondroprogenitors, *Col2a1^+^* early-stage chondrocytes, and some *Acan^+^* chondrocytes (fig. S3D). Each differentiation stage spread throughout the mesenchymal projection (Fig. 1C, brown metacells). Dense regular connective tissue progenitors differentiating into tenocytes and ligament fibroblasts showed expression for *Col3a1*, while more differentiated tendon progenitors expressed *Scx* (fig. S3E). Noteworthy, *Col3a1* transcripts were also detected in the most differentiated chondrocyte metacells. The remaining uncommitted mesenchymal cells expressed none of the canonical chondrogenic or connective tissue markers. This undifferentiated mesenchyme exhibited a diversity of cell states that could be classified into two main classes based on *Lhx2* and *Hand2* expression (fig. S3F). At this level of resolution, we did not capture any transcriptional difference along the dorsal-ventral (DV) axis in the mesenchyme (fig. S4, A and B), and although our clustering allowed separating the AER from the dorsal and ventral ectoderm, we were not able to separate the dorsal *Wnt7a*-expressing ectoderm from the *En1*-expressing ventral ectoderm (*36*) (*En1* is also expressed in the AER; fig. S4C). Two metacells representing cell states derived from the same cell type often show multiple differentially expressed TFs (log fold change > 2). This highlights the existence of distinct regulatory programs within a given cell type, corresponding to differentiation stage, spatial position, or other factors (fig. S5).

### The fine-scale organization of limb bud patterning

**Fig. 2.**
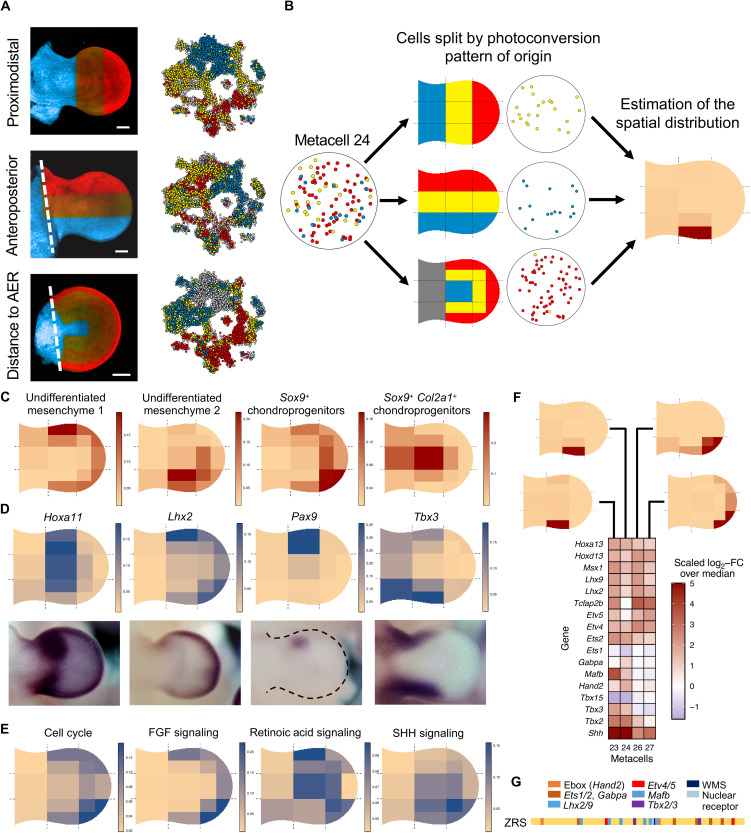
Integrating TATTOO-seq data to build a spatial map of cell state and gene expression. (**A**) Maximum intensity projection of z-stack images of each of the three photoconversion patterns. (**B**) Strategy to assemble information from different patterns of photoconversion to infer the position of cell states. (**C**) Spatial distribution for some aggregated mesenchymal cell types. (**D**) Examples of vISH obtained ab initio from TATTOO-seq data. Images were obtained from the EMBRYS database (*79*). (**E**) Spatial projection of summarized gene expression for relevant gene sets. (**F**) Spatial and transcriptomic dissection of the *Shh*-positive ZPA. FC, fold change. (**G**) Schema of the ZRS enhancer–regulating *Shh* and its cohort of TF-binding sites.

As mentioned above, the flexibility of optical registration allows using patterns that closely fit the spatial organization of the structure of interest and capture its multidimensional organization by different user-defined patterns of photoconversion. For the limb, we applied patterns of photoconversion to register cell positions along the PD and AP axes of the limb bud, with respect to the distance to the AER (Fig. 2A). The gating strategy is shown in fig. S6. Overlaying this color information about the metacell graph revealed a strong spatial component to its organization (Fig. 2A). With the exception of endothelial and ectodermal metacells, metacells’ color composition was more homogeneous than expected by chance (fig. S7), indicating that metacells reside at precise spatial locations. This fact supports that metacells represent different biological states and are not clustering artifacts. It directly demonstrates that cell position is a strong component of mesenchymal cell identity as reflected by the expression of position-specific transcriptional programs.

Although the information provided by a single cell is limited to one axis of our coordinate system, metacells comprise cells from all three photoconversion axes. Each metacell can thus be positioned into a more complex 14-bin spatial grid (Fig. 2B and fig. S8; see Materials and Methods) by calculating the spatial probability distribution that maximized the likelihood of observing a metacell’s color composition. Individual metacells’ probability distributions were aggregated by cell type annotations, resulting in cell type spatial distributions (Fig. 2C).

This spatially resolved atlas showed that transcriptionally related metacells can have markedly different spatial distributions and allowed for the exploration of gene expression with increased precision and resolution. For each expressed gene, we derived a virtual in situ hybridization (vISH) pattern by computing the probability distribution over space for a random UMI of that gene to be found in each spatial bin. vISH results closely matched actual in situ hybridization experiments (Fig. 2D and fig. S9). We provide access to a precomputed atlas of vISH for ~17,000 mouse genes at http://nobelmarlowlab.uchicago.edu:8888/TATTOOseq_vISH/. vISH further enabling to visualize genes and pathways across cell types at new depth and precision. We not only can readily compare the expression of two or more genes (fig. S10A) but can also break down gene expression patterns by cell type (fig. S10B). Process-specific gene expression profiles can also be combined to examine the spatial distribution of integrated pathways or biological processes, such as cell proliferation or signaling pathway-responsive cells (Fig. 2E).

As an example, we used vISH to map the ZPA, which organizes limb patterning along the AP axis (*1*, *26*). Its defining gene, *Shh*, was robustly expressed in four metacells *Shh* (23, 24, 26, and 27) (Fig. 2F) which collectively mapped to ZPA at the limb’s posterior margin. Metacells 23 and 24 were located medially, whereas metacells 26 and 27 were located more distally, suggesting that they may represent previously unknown ZPA subdomains. As mentioned above, metacells 26 and 27 are likely to represent the same general state, and the differences are the possibility of consequences of a batch effect or a stage difference. Levels of *Tbx2* expression constitutes the main difference between distally located metacells 26 and 27, which is consistent with the distal extension of the *Tbx2* expression domain throughout the hand plate formation and the possible earlier stage of development of the cells assigned to metacell 27. Several TFs showed differential expression between metacells 23 and 24 and 26 and 27 (Fig. 2F and fig. S11), including *Hand2*, *Hox*, and Ets TFs, which have been shown to regulate *Shh* expression (*37*–*39*). Conserved binding motifs for these TFs are present within the zone of polarizing activity regulatory sequence (ZRS), the *Shh* limb enhancer (Fig. 2H) (*4*, *23*, *40*). The existence of different transcriptional regimes between the proximal (represented by metacells 23 and 24) and distal (represented by metacells 26 and 27) parts of the ZPA, including ZRS-associated TFs, raises the possibility that *Shh* may use different modes of regulation in different parts of the ZPA. On the proximal side, its activity may be primarily driven by *Tbx2/3* and *Hand2*, whose expression is higher proximally. Whereas distally, other factors (*Hoxd13*, *Tfap2b*, and ETS TFs) may play a relatively increased role. Supporting the multiplicity of ZRS-associated complements of TFs, mutations in the ZRS have been shown to affect its activity to different degrees along the PD axis (*39*). It should be emphasized that these different regimes of regulation of *Shh* through its ZRS enhancer, as proposed here, do not necessarily imply the partition of the ZPA into distinct domains. These regimes may coexist within the ZPA, with their relative contribution to *Shh* expression varying along the PD axis (23/24 versus 26/27) and possibly the AP axis (23 versus 24). This hitherto hidden diversity of regulatory regimes within the ZPA may contribute to the diversity of limb morphologies resulting from genetic variants in the ZRS or ZRS-associated TFs as they may modulate the shape and signaling strength of the ZPA (*41*–*43*).

### A TF code of spatial position

**Fig. 3.**
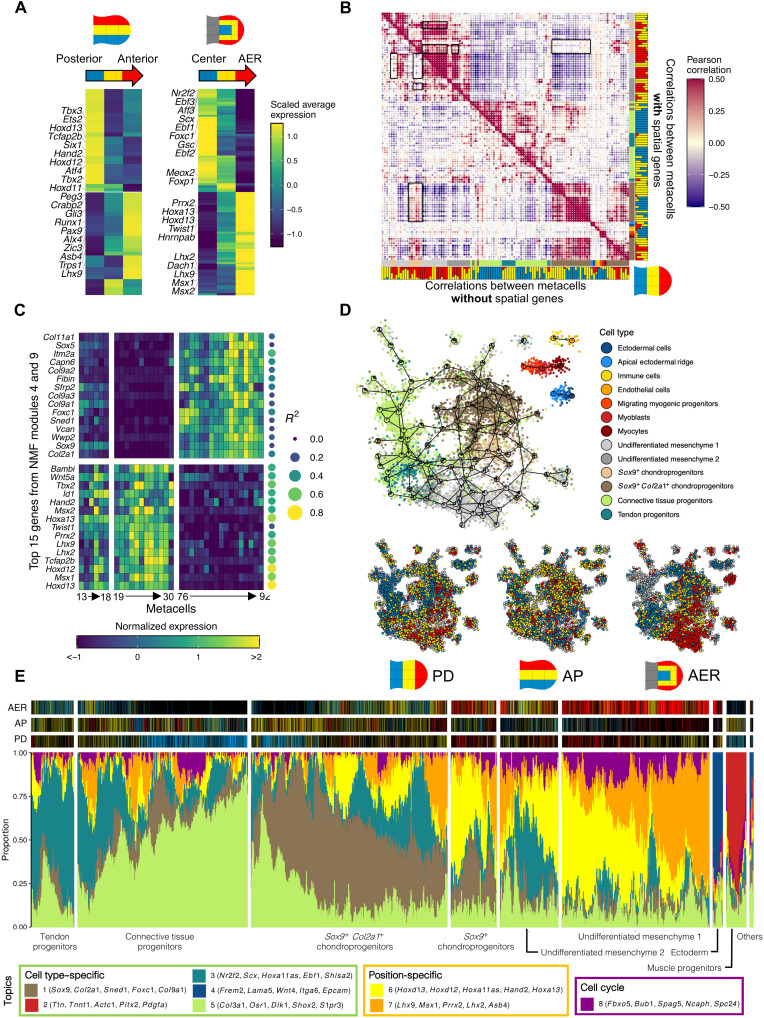
Deconvoluting positional information and cell type–specific regulatory programs. (**A**) Average expression per spatial compartment for TFs that exhibit spatial trends in the mesenchyme. Top 10 TFs with most significant positive and negative Spearman correlation values are annotated. (**B**) Pairwise correlation heatmap for metacells. Top: Using all genes defined as HVGs. Bottom: Excluding spatial genes. Cell types are indicated using the same color code as for Fig. 1 and color composition for the PD photoconversion pattern. (**C**) Normalized expression of the top 15 genes (by weight) in NMF modules 4 and 9 across metacells 13 to 30 and 76 to 92. For each gene, the *R*^2^ of the spatial regression is indicated. (**D**) Two-dimensional projection after clustering the TATTOO-seq dataset with no spatial genes. Top: Cells are colored by annotation. Bottom: Cell colors for each photoconversion pattern. (**E**) fastTopics structure plot. Top: Cell colors for each pattern (black if the cell originates from a different pattern of photoconversion).

We next sought to use the TATTOO-seq atlas to comprehensively identify TFs whose expression exhibits strong spatial trends in the medial and distal mesenchymal cells. Of 1390 TFs detected in the autopod and zeugopod mesenchymal metacells (>100 UMIs in total), 202 showed variable expression (≥1 metacell with expression of >1.5 × median expression across all mesenchymal metacells). We used Spearman’s rank correlation to assess monotonic trends in gene expression as a function of the distance to the AER and along the AP axis (Fig. 3A). We uncovered a multitude of TFs whose expressions are correlated with position, highlighting the spatial heterogeneity of regulatory states in the limb bud. TFs forming anterior-to-posterior gradients not only included previously known genes, such as *Alx4*, *Asb4*, and *Zic3* (*31*), but also revealed new genes with unknown functions in limb development (*Thra*, *Lmo4*, and *Cited2*). Twenty-six TFs formed posterior-to-anterior gradients of expression, comprising the 5′-*Hoxd* genes (*Hoxd10* to *Hoxd13* and *Evx2*) and *Hand2*. Forty-nine TFs were expressed close to the AER, including many of the known targets of AER-secreted FGFs and bone morphogenetic proteins (BMPs) (*Lhx2/9*, *Msx1/2*, and *Etv4*) and distal *Hox* genes (*Hoxd13* and *Hoxa13*). A total of 104 genes, including genes involved in connective tissue differentiation (*Sox5/6/9*, *Scx*, and *Runx1/3*) (*29*, *44*–*46*) showed the opposite trend, with weaker expression underneath the AER.

### Deconvoluting spatial patterning and cell differentiation programs

As an attempt to deconvolute the spatial and cell type–specific regulatory logics operating in the mesenchyme, we sought to classify genes as primarily carrying “spatial” or “cell type”–related information. We reasoned that genes encoding spatial information would have a similar spatial distribution of expression across cell states, while genes regulated in a strictly cell type–specific manner would show poor predictability from metacell position. We used linear regression to predict gene expression from the spatial probability distribution of each metacell for all genes and computed overall significance using *F* test. After Bonferroni correction for multiple testing, we retained models for 4713 genes (of 17,537, adjusted *P* < 0.01). These genes include major components of limb signaling pathways (table S2). We then compared two pairwise correlation matrices between metacells computed using either all highly variable genes (HVGs) or only non–“spatially regulated” HVGs (Fig. 3B). We noted two types of changes: The correlation between two groups of distal metacells decreased without spatial HVGs, while the correlation between one of these groups and proximal chondrogenic metacells increases. Nonnegative matrix factorization (NMF) of the gene-by-metacell expression matrix revealed gene modules that could explain these changes. NMF module 4 exhibited high weights for known patterning genes with strong spatial regulation (14 of 15 top genes with *R*^2^ > 0.45), while module 9 exhibited high weights for chondrogenesis genes with overall low spatial regulation (Fig. 3C). The exclusion of spatially regulated genes to compute correlations decreased the influence of module 4 and, therefore, the similarity between metacells that simply reside at similar positions. It also unmasked a high similarity between chondrocytes in the distal and proximal compartments, which also appeared if single-cell data are clustered using only nonspatially regulated genes (Fig. 3D and fig. S12). This new graph projection grouped cells together by cell types, reducing the notable position-driven dispersal of the initial projection (Fig. 1C).

To further investigate the interplay between cell position and cell fate, we used fastTopics (*47*, *48*) to model each cell as a mixture of different (*k* = 8) topics, describing cell states as combinations of more or less independent modules (Fig. 3E). This uncovered a spectrum of combinatorial topic utilization consistent with the continuum of cell states highlighted by MetaCell. Notably, two anticorrelated topics reflected positional information in the undifferentiated mesenchyme 1 (topics 6 and 7). The relative importance of these topics followed a medial-anterior to distal-posterior axis, showing the strong correlation of these two topics with spatial position. These spatial topics 6 and 7 were also widely expressed at different levels in chondrogenic progenitors, providing position-associated diversity to cells otherwise defined by the core chondrogenic topic 1.

### Integration of spatial information by position-specific regulatory landscapes

As chondrogenesis is initiated as the limb develops along the PD axis, chondroprogenitors at different stages of their differentiation lie in different spatially regulated transcriptomic subspaces, which is heavily reflected in the single-cell clustering and may mask lineage relationships between cells located at different positions. To compare chondrocyte differentiation trajectories in different PD compartments, we constructed a single-cell graph of chondrogenesis in a stepwise manner, using the subspaces defined for groups of cells originating from consecutive spatial positions, adapting an approach used to reconstruct cell trajectories across time series (fig. S13A) (*49*). By allowing us to compare chondrogenesis gene expression despite the confounding effect of spatial compartmentalization, this method showed that spatially segregated chondrocytes display overlapping core chondrogenic transcriptomic states. Further analysis suggested that spatially segregated chondrocytes follow a largely stereotypic differentiation trajectory marked by the same core genes (fig. S13B).

**Fig. 4.**
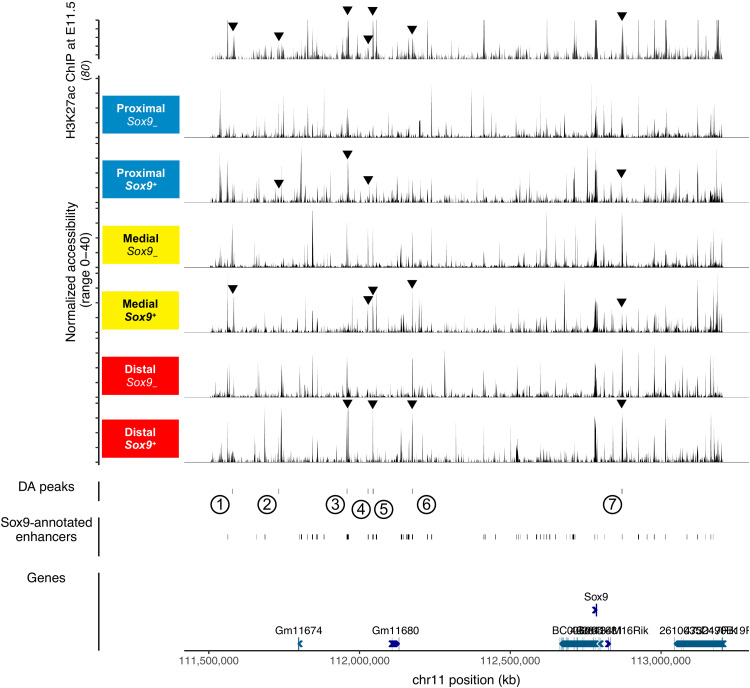
Putative spatial regulation of the accessibility landscape at the *Sox9* locus. Cell identity and position were transferred from our TATTOO-seq data to limb scATAC-seq data (*54*) using Seurat’s TransferData function. All peaks in the *Sox9* regulatory domain (chr11: 111,503,853 to 113,206,397, mm10) were tested for differential accessibility. The top track shows the H3K27ac chromatin immunoprecipitation (ChIP) sequencing signal in E11.5 whole limb buds (two replicates were aggregated) from (*80*). Differentially accessible (DA) peaks are indicated and previously described *Sox9* enhancers (*56*).

Among those genes, *Sox9* is a key regulator of chondrogenesis (*50*). It shows a dynamic and complex regulation during limb development (*51*), involving a large regulatory landscape extending over 2 Mb and comprising dozens of potential limb enhancers (*52*, *53*). To assess whether this landscape was identically activated along the limb PD axis, we leveraged published wild-type E11.5 limb bud single-cell assay for transposase-accessible chromatin sequencing (scATAC-seq) data (*54*) to identify chromatin-accessible elements. We used Seurat (*55*) to transfer the position along the PD axis and *Sox9* expression levels from our TATTOO-seq dataset to the scATAC-seq data and generated pseudo-bulk ATAC profiles based on position and *Sox9* expression (Fig. 4). Despite the limited number of cells in each category (<300 distal *Sox9*^+^ cells), we were able to detect seven ATAC peaks (*P* < 0.05; table S3) in the *Sox9* regulatory domain/topologically associating domain that are differentially represented along the PD axis (Fig. 4), five of which correspond to previously described *Sox9* enhancers (*56*). While preliminary, these data suggest that spatial information could be relayed to cell-fate controlling genes via position-specific enhancers, enabling specific modulation of other stereotypic differentiation regulatory networks.

### High-resolution characterization of cell fate and patterning alteration in mutant limbs

**Fig. 5.**
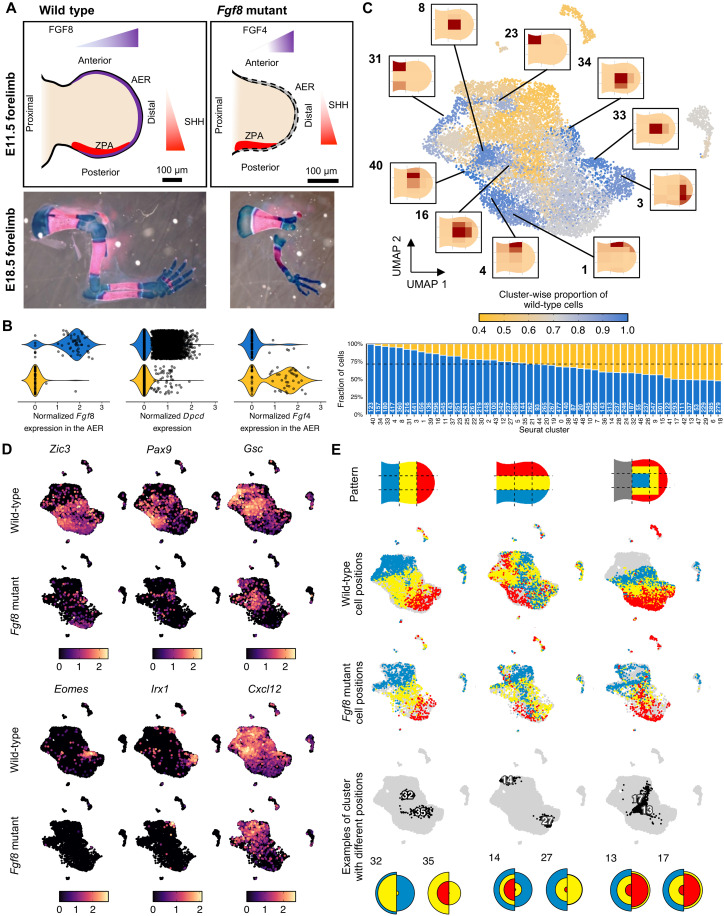
Using TATTOO-seq for high-content phenotyping of a patterning mutant. (**A**) Top: Schematic representation of the E11.5 wild-type and mutant forelimb buds. Bottom: Skeletal preparation of wild-type and mutant forelimbs (Alcian blue/alizarin red). (**B**) Expression of *Fgf8*, *Dpcd*, and *Fgf4* for each genotype. (**C**) Top: Uniform Manifold Approximation and Projection (UMAP) of the combined wild-type and mutant datasets colored by cluster-wise fraction of wild-type cells. Bottom: The fraction of wild-type cells. (**D**) UMAP showing marker gene expression for mutant cell–depleted clusters. (**E**) Top: UMAP showing cell colors for each photoconversion pattern split by genotype. Bottom: Color distribution in some Seurat clusters for each genotype (left, wild-type mutant; right, *Fgf8* mutant).

Patterning defects can lead to complex phenotypic consequences by perturbing cell differentiation programs, as well as their spatial modulation and coordination. We sought to apply TATTOO-seq to assess how limb patterning and differentiation are reorganized when positional information is perturbed. FGF8 is the main FGF molecule controlling the growth and patterning function of the AER (*27*). DEL(PolL-SHFM) (*57*) mice carry a deletion of the main *Fgf8* enhancers in the AER and fail to express *Fgf8* in the AER. They display shortened bones and lack proximal and anterior skeletal elements (humerus, radius, and digits 1 and 2) (Fig. 5A). To examine the molecular and positional changes that ultimately lead to the observed malformations, we produced a TATTOO-seq atlas for E11.5 DEL(PolL-SHFM) homozygous forelimb buds (embryos, *n* = 3; limbs, *n* = 4). The gating strategy (fig. S14) used for the mutant samples is the same as that of wild-type samples, but since mutant limbs are shorter, the AER pattern was applied to the whole limb, including the proximal cells. After filtering, we obtained high-quality transcriptomes for 3349 mutant cells. To compare the cell populations and their transcriptome between wild-type and mutant limbs, we produced a coarser joint clustering using Seurat (*55*), as the high-resolution clustering provided by MetaCell places mutant and wild-type cell in different metacells. As a control, we first examined the expression of genes studied by single-gene in situ hybridization experiments in *Fgf8* mutants (*27*, *57*). In our dataset, we found that *Dpcd*, a bystander gene included in the DEL(PolL-SHFM) deletion, was almost undetectable in the mutant cells (Fig. 5B). Furthermore, as expected, *Fgf8* expression was abolished in the mutant AER cluster, while we detected a compensatory up-regulation of *Fgf4*, as reported previously (*27*).

We then investigated how reduced AER function affected the presence of the different cell types, their transcriptomes, and spatial distributions. The average gene expression for each Seurat cluster (both aggregated and split between by genotype) is indicated in table S4. The comparison of stage-specific marker expression with E10.5 data did not suggest a simple developmental delay (fig. S15). We first determined the relative fraction of wild-type and mutant cells for each cluster (Fig. 5C). Although we could not unambiguously calculate the expected cluster-wise fractions under the null hypothesis (AER pattern samples were enriched for distal cells, by up to twofold), several robust differences could not be explained by this limited sorting bias. At this level of resolution, we did not detect mutant-specific cell states. However, several wild-type cell states were almost absent from the mutant samples (Fig. 5C). Many depleted clusters (expressing *Zic3* distally, *Pax9* medially, and *Gsc* proximally) were located in the limb’s anterior compartment (Fig. 5D), which likely explained the absence of anterior skeletal elements at later stages. However, we also noted that posteriorly located cell states (e.g., cluster 3) and some secondary signaling centers for connective tissue differentiation defined by *Cxcl12* and *Fgf18* (*58*, *59*) were strongly diminished in the mutant. Examining chondrogenic cell states, we observed that although chondrogenesis appear to proceed overall normally in the mutant, the reduction in distal FGF signaling led to a reduced diversity of spatially distributed chondrogenic cell states: We found fewer distal *Sox9*-positive cell states expressing *Eomes* [base of digit 4 (*60*)] and *Irx1* [tip of digits 2 to 4 (*61*)] (Fig. 5, C and D). These distal chondrogenic cell states have been proposed to act as regulation centers expressing different BMP pathway components involved in joint formation, including *Gdf5*, *Inhb*, *Nog*, and *Chrdl1* (*62*–*65*). Although the depletion of these cell states is only partial, no expression of these signaling-related genes was detected in the mutant data (fig. S16A), which could account for the missing elbow joint in mutant limbs (*27*, *57*). To facilitate the exploration of those cell states, fig. S17 shows the contribution of each wild-type metacell to each Seurat cluster.

Besides changes in specific cell state populations, we also identified a pervasive adaptation of the transcriptional states of otherwise conserved cell types, with the expression of multiple genes being affected by directly or indirectly by the reduction of *FGF* signaling from the AER (fig. S16C and table S5). Regarding the spatial distribution of the different limb cell states, only a few positional changes were detected along the PD and AER axes, and most of the spatial rearrangements were observed along the AP axis (Fig. 5E and fig. S18). Consistent with the absence of most anterior mesenchyme cell states, mutant cell states exhibited relatively more anterior colors than their wild-type counterparts. However, we also identified some cell states that were located more posteriorly in the mutant than in the wild-type, indicated complex alterations of patterning information, maybe resulting from the effect of reduced *FGF* signaling on the maintenance of ZPA activity and reduced *Shh* signaling (*66*, *67*).

## DISCUSSION

siTATTOO-seq constitutes a simple, robust, and flexible approach to integrating spatial position and single-cell transcriptomes. The data and analyses provided here illustrate how TATTOO-seq can help making progress toward an integrated and high-resolution description of the dynamic processes involved in embryonic patterning. Orthogonal analyses such as NMF, topic modeling, and graph manifold assembly on TATTOO-seq data reveal the respective contributions of patterning and differentiation-specific gene modules to cell states, identify specific TFs that mediate spatial identity, and constitute a first step in disentangling spatial and cell type–specific gene regulatory programs. We show that positional information substantially modulates the transcriptome of limb chondrocytes and their core differentiation program and suggest that this core differentiation program operates at distinct positions through different, context-specific, modalities, which associate position-specific TFs to position-specific cis-regulatory elements. These differentially activated enhancers may act as specific positional integrators for common differentiation pathways and might be primary targets for regionalized natural and pathological variations in morphology (*68*). TATTOO-seq further offers a new integrative and multilevel description of developmental defects, as illustrated by the complex cellular, molecular, and spatial changes revealed in the *Fgf8-*mutant limb buds. We expect that such a comprehensive, spatially resolved molecular data could provide a much finer-grained representation of “mutant phenotypes” and reveal how genetic or environmental perturbations may propagate through developmental gene regulatory networks and rewire them.

TATTOO-seq requires only standard equipment, namely, a confocal microscope and an FACS. While coupled here to a microplate–based single-cell technology, it can easily be adapted to other protocols, including droplet-based protocols, for example, using cell multiplexing/hashing strategies to label sorted cells of the same color. TATTOO-seq shares conceptual similarities to Tomo-seq (*69*), although it provides single-cell resolution and the simplicity and rapidity of optic labeling over physical sectioning. While the approach as shown here can be used on thick samples (up to 500 μm), the shape of the sample and, ideally, its patterning geometry must be compatible with the flat geometry of the microscope. For nonflat structures, the use of thick vibratome sections is recommended.

Because our approach relies on in silico cell aggregates, instead of individual cells, it is suitable for relatively sparse datasets and works without imputation. TATTOO-seq does not require spatial landmark reference genes nor does it assume the homogeneity of gene expression at a given position, which are strong limitations of retrospective mapping (*9*, *70*) or pseudo-spatial ordering (*71*). Thus, it is immediately suitable for the analysis of mutant phenotypes without the need to build a mutant reference map of gene expression.

Although the spatial resolution used in this study is relatively coarse (50 to 100 μm, depending on the photoconversion pattern), this resolution is in par with other spatial transcriptomics methods such as Slide-seq (*15*) or the commercial Visium platform (10x Genomics), which are based on barcoded beads or spots covering 50- to 100-μm regions of a tissue section. While recent progress in bead/spot arraying can theoretically offer a resolution of 10 μm (*72*), these approaches do not offer true single-cell resolution, are still better suited to identify the spatial distributions of single-cell clusters acquired separately, and may come at a much higher cost. In comparison, our approach could resolve finer spatial grids as specified by the user, since it is only limited by the precision of the photoconversion and the capacity of flow cytometry to detect graded fluorescence levels. However, as the number of relative fluorescence levels that can be reliably separated is not infinite (at least four with our current set up), a higher spatial resolution comes with a reduction of the area that can be studied. While a genetically encoded photoconvertible protein is at the basis of our method, the recent development of photoconvertible and clickable dyes or the use of photoconvertible membrane labeling dyes (*73*, *74*) may allow further implementation of TATTOO-seq–based approaches in nontransgenic and nonmodel organisms or in patient-derived samples.

## MATERIALS AND METHODS

### Animals

Transgenic CAG-KikGR-1 mice (*32*) (KikGR1 hereafter, gift of A. Aulehla) were maintained inbred and genotyped either by polymerase chain reaction (PCR) using internal primers (p1, GAAATGAAGATCGAGCTGCGTATGG; p2, CACCCTTCAGCACTCCATCACGCAC) and a standard program with 65°C annealing temperature or by assessing green fluorescence in distal phalanx biopsy. DEL(PolL-SHFM) mice (*57*) were maintained on an inbred C57BL/6J background.

Homozygous KikGR1 males and wild-type females were crossed to generate control embryos. For mutant analysis, KikGR1/+; DEL(PolL-SHFM)/+ males and DEL(PolL-SHFM)/+ females were crossed to generate experimental and control embryos. E0.5 was defined as noon of the day when the vaginal plug was detected. Embryos were collected at E11.5 and dissected in ice-cold phosphate-buffered saline (PBS) supplemented with magnesium and calcium chloride. KikGR1 heterozygous embryos displayed strong and widespread green fluorescence in all tissues at all observed stages. All animal procedures were approved and performed accordingly to the ethical principles and regulatory guidelines and protocols set up by the Institutional Animal Care and Use Committees at the Institut Pasteur and the University of Chicago.

### Photoconversion and imaging

Samples were mounted in PBS supplemented with magnesium and calcium chloride between a glass slide and a coverslip separated by several layers of adhesive tape. Photoconversion was performed, and all fluorescence images were acquired using a Zeiss inverted confocal microscope (LSM 800) using a 10× objective. Green and red fluorescence images were obtained by excitation with the 488-nm (1 mW, gain = 580, digital gain = 2) and 561-nm (1 mW, gain = 580, digital gain = 2) laser diodes, respectively. The spectra of enhanced green fluorescent protein and mCherry were used for nonphotoconverted and photoconverted version of KikGR1, respectively, and adjusted such that they did not overlap. For green-to-red photoconversion of kikGR1 proteins, the 405-nm laser diode was used with variable power (100% laser power was equal to 5 mW). Full conversion (red color) was obtained after four iterations at 40% power, while partial conversion was obtained after four iterations at 10% power (for the PD pattern), 9% power (for the AP pattern), or 10 and 6% power (for the four-color AER pattern). For the photoconversion along the PD and AP axes, the size of the photoconverted regions was determined to be one-third of the length of the limb bud (from the tip to the flank or from the anterior-most part to the posterior-most part, respectively) as measured with the ZEN software. For the AER axis, the photoconverted regions were determined to be approximately one-fourth of the radius of the dissected autopods and zeugopods. While we could easily create 4° of photoconversion and separate them by flow cytometry, we grouped the two red-most colors into one color bin to increase the number of cells. We always applied the partial photoconversion (9 or 6% power) to both the “partially” and “fully” photoconverted target regions to ensure that cells at the border between the two regions are photoconverted to the same degree as the fully photoconverted region. To maximize the homogeneity of the photoconversion, we measured the thickness of the limb and chose the plane of photoconversion to coincide with the middle of the limb. The total duration of the photoconversion process was ~3 min in our particular setup. E10.5 samples were processed identically to E11.5 samples.

### Tissue dissociation and FACS

Limb buds were dissected and incubated for 5 min in 270 μl of Liberase (0.22 Wunsch unit/ml; Roche, 5401119001) in EDTA-free, calcium-free, and magnesium-free PBS with agitation (900 rpm) at 37°C in a low-binding microcentrifuge tube (Biozym, 710176). Mechanical disruption was then performed by pipetting through a p200 gelatin-coated tip. The enzyme was inhibited by adding 30 μl of 0.5 M EDTA, and volume was adjusted to 1 or 1.5 ml with calcium- and magnesium-free PBS. While this method allows the efficient recovery of mesenchymal cells, it is not optimized to recover ectodermal cells. Tight junctions could show limited sensitivity to short-duration collagenase treatment, thereby explaining why we recovered few ectodermal cells.

Calcein Violet AM (1 μM; Life Technologies, C34858) and 5 nM Sytox Red (Thermo Fisher Scientific, S34859) from Invitrogen were used as live and dead cell markers for the sorting, respectively. Excitation and emission spectra were well separated from those of the nonphotoconverted and photoconverted KikGR1. Single-cell suspensions were filtered through the 35-μm BD Falcon Cell-Strainer Cap (352235) into an FACS tube, which was maintained on ice before sorting and at 4°C during the experiment in the injection chamber. Sorting was done on a BD FACSAria III with a flow rate at the lowest value (“1.0”). A mild spectral compensation was applied to phycoerythrin-allophycocyanin (PE-A) (red) and Violet-F-450/40-A (violet) channels from the fluorescein isothiocyanate–A (FITC-A; green) channel.

Debris and doublets were excluded from the analysis using a sequential gating strategy relying on first side scatter (SSC-A) versus forward scatter (FSC-A), followed by FSC-H versus FSC-W. Live cells were selected on the basis of live/dead staining gating on Violet-F-450/40-A (Calcein Violet AM) versus allophycocyanin-A (Sytox Red). Only Calcein-positive Sytox-negative cells were included in the analysis. Cells showed similar viability percentages regardless of the mounting and photoconversion status (~90 to 97%). Cells with a low fluorescence value in both green (FITC-A) and red (PE-A) channels were also excluded. No further binning on red and green fluorescence is performed. Cell colors are inferred a posteriori as described below (see the “Identification of cell colors” section). E10.5 samples were processed identically to E11.5 samples.

### Massively parallel scRNA-seq

Cells were processed by MARS-seq as previously described (*33*). Briefly, live single cells were sorted into 384-well capture plates containing lysis solution and barcoded poly(T) reverse transcription (RT) primers. UMI barcodes contain a cell-specific/well-specific label and an 8–base pair random molecular tag (RMT). After evaporation of the lysis buffer, the RT reaction was performed in the presence of External RNA Controls Consortium (ERCC) RNA Spike-in (Ambion). Unused RT primers were digested using Exonuclease I [New England Biolabs (NEB)], and wells were pooled by half plates. Second-strand cDNA synthesis was then performed, and products were in vitro transcribed overnight using T7 polymerase (NEB) for linear amplification. After RNA fragmentation, a partial Illumina Read1 sequencing adapter containing a plate-specific barcode was single strand–ligated using T4 RNA ligase I (NEB), and the product was reverse-transcribed. Last, the product was purified and PCR-amplified by PCR (14 cycles) with primers containing the Illumina P5-Read1 and P7-Read2 sequences. Concentration of the purified library was assessed with a Qubit fluorometer (Life Technologies), and mean molecule size was determined with a 2200 TapeStation instrument (Agilent Technologies). Libraries were pooled and paired-end sequenced using an Illumina NextSeq 500 sequencer at a median depth of ~45,000 reads per cell. Read1 was 70 nt long and covered the plate-specific barcode and the cDNA. Read2 was 16 nt long and covered the UMI and well-specific label. Raw and processed data sets are available from the Gene Expression Omnibus (GEO) repository (GSE202326).

### Quality check and read mapping

Reads were demultiplexed and count tables were built as described in (33) (scripts are available at https://github.com/tanaylab/MARS-SEQ and https://doi.org/10.5281/zenodo.7316279). Cell-specific/well-specific labels and RMTs were extracted. Reads with low-quality (Phred < 27) barcodes were discarded to prevent ambiguous or spurious assignments of reads to cells or unique molecules. Read2 reads whose cell-specific or well-specific labels were unknown or mutated were discarded. Read1 reads were mapped to the mouse genome (mm9) using Ensembl gene annotations (downloaded in July 2017) using Bowtie2 (*75*).

### Metacell analysis

We clustered the wild-type E11.5 dataset using MetaCell (*35*). Briefly, an ideal metacell is a group of cells whose expression profiles are statistically equivalent to independent sampling from a single underlying transcriptional state. This is achieved by first creating a *k*-nearest neighbor (k-NN) graph of the individual cells based on expression similarity and by partitioning this graph into a disjoint sets of cells, metacells, which are both small (~100 cells each) and as close to the above ideal metacell as possible.

Because plates were processed in two batches, we filtered out cells with fewer than 2000 or 2500 UMIs (lower-quality transcriptomes) or more than 15,000 UMIs (potential doublets). We excluded mitochondrial genes (annotated with the prefix “mt-”) from the analysis. Variable genes were selected using the parameters Tvm = 0.1, Ttot = 50, and T_top3 = 3. We then excluded cell cycles, histone genes, ribosomal genes, small nuclear riboprotein genes, some long noncoding RNA (annotated with the suffix “Rik”), and poorly supported gene models (annotated with the prefixes “RP-,” “Gm,” and “AC”) from the variable genes. In addition, we identified genes that showed a batchy expression. To do so, we performed a first clustering, and for each metacell with a sufficient number of cells from both batches (>15), we repeatedly (10 times) randomly sampled and aggregated UMIs from 10 cells (UMIs from individual cells were downsampled to eliminate the effect of sequencing depth). If the median fold change for a gene was >1.6 in at least one metacell, then the gene was considered batchy and blacklisted for feature selection (but not omitted from the analysis). When excluding spatial genes, we additionally blacklisted genes with adjusted *P* < 0.01 for the linear regression. A k-NN graph was built using Pearson correlation and *k* = 100, and 500 bootstrap iterations were performed (0.75 resampling probability). Metacells were built with a minimum size of 20, *k* = 30, and α = 3. Inhomogeneous metacells were split, and outlier was removed (Tlfc = 3). Otherwise, default parameters were used.

### Identification of cell colors

To infer a single-cell color from the intensities of the PE (red, photoconverted) and FITC (green, nonphotoconverted) channels, we examined the ratio between the log of these intensities

$$s=\frac{\log_{2}\mathrm{PE}}{\log_{2}\mathrm{FITC}}$$

The distribution of this ratio exhibited the expected number of peaks (3 or 4) across all samples, although the exact location of the peaks and their separation varied. We therefore modeled the distribution of each individual limb separately as a mixture of skewed normal distributions whose parameters were estimated using an expectation-maximization algorithm implementer in the R package mixsmsn (*76*) (“smsn.mix”, family = “Skew.normal”, nu = 3). Three components were used for the PD and AP patterns, and four were used for the AER pattern. The color of each individual cell was determined using the posterior probabilities. For the AER pattern, we merged the two red-most compartments (high ratio-of-log values) to increase the number of cells in that category.

### Modeling the TATTOO-seq photoconversion process

We treated the limb-bud as a two-dimensional shape, constructed through concatenation of Bézier curves. We then assumed that the AP and PD photoconversion patterns split this shape into three regions of equal length along the relevant axis, whose fluorescence is mostly green, mostly yellow, or mostly red. On the basis of pictures of the photoconverted limb buds, we estimated that the part of the limb bud that was dissected for AER pattern photoconversion roughly matched the yellow and red regions of the PD pattern. We then approximated the boundaries of the AER photoconversion regions using further concatenations of Bézier curves, choosing the parameters that best fit the pictures. Note that although the AER photoconversion split the limb bud into four regions, we combined the two red-most regions in our model, as the number of cells collected that had red-most fluorescence was extremely low.

We then created a generative model connecting a cell’s original position within the limb bud with its measured fluorescence. We split the modeled limb bud’s shape into distinct spatial bins (referred to henceforth as sbins). Given the sbin containing the cell and the pattern that was used for photoconversion, it is possible to infer a distribution over the cell’s fluorescence being measured as green, yellow, or red. It is also possible to infer the probability that the cell will be completely missed by the experiment (as is the case with proximal cells when the AER photoconversion pattern is used).

Theoretically, on the basis of the above photoconversion model, it is possible to split the limb bud into 17 sbins, which set a single, deterministic fluorescence color under all photoconversion patterns. However, using these sbins to infer cell positions led to extremely poor results, probably due to the failure of such a strictly deterministic model from handling slight deviations between the model and the experimental reality (for example, the limb bud’s shape and the exact dissection line differed slightly between embryos). Instead, we split the limb bud into 36 sbins based on a square 6 × 6 equidistant grid along the AP-PD axes. Within each such sbin, the fluorescence color under the AP and PD colors is deterministically defined, while the distribution of fluorescence under the AER pattern is assumed to be proportional to the area of the sbin that is modeled to be photoconverted to green, yellow, and red, respectively. We then merged together sbins whose distributions were highly similar, ending up with 14 sbins.

### Inferring cell positions

The fluorescence information provided for each cell by TATTOO-seq only limits its position to one of three or four broad spatial regions. To gain a finer spatial resolution, we used the reproducibility of patterning of the mouse embryo system. This allowed us to pool together spatial information collected across multiple limb buds and multiple photoconversion patterns and infer a distribution over the limb bud’s sbins.

We will mark by λ one of the photoconversion pattern, i.e.

λ∈{AER,AP,PD}

Given a subset of cells, we can calculate the regularized, empirical distribution of the measured fluorescence under each of the pattern λ

$$P_{\mathrm{emp},\lambda }(F)$$

We assumed that the subset of cells defines some transcriptional criterion *M* (e.g., a cell type) that implies a distribution over the model’s sbins. Then, the sbins containing cells belonging to *M* will be distributed according to

$$P_{\theta ,\lambda }(S|M)$$

where θ is a parameter. Last, the photoconversion model described in the previous section implies a distribution over the measured fluorescence given the sbin and the photoconversion patterns

$$P_{\lambda }(F|S=s)$$

Given the above, we can calculate the expected fluorescence of cells belonging to *M*

$$P_{\theta ,\lambda }(F|M)=\sum_{s}P_{\lambda }(F|S=s)\cdot P_{\theta ,\lambda }(S=s|M)$$

To infer the correct value of θ, we minimize the Kullback-Leibler divergences between the inferred distributions and the empirical ones

$$\theta =\mathrm{argmi}n_{\;\theta }\sum_{\lambda }D_{\mathrm{KL}}[P_{\theta ,\lambda }(F|M)||P_{\mathrm{emp},\lambda }(F)]$$

As the distributions *P*_θ, λ_(*S* ∣ *M*) are discrete, the parameter θ can be taken to be the complete definition of the distribution. To solve the minimization problem, we used a constrained interior point algorithm, implemented in the scipy.optimize.minimize() function (from the scipy Python package) with a method parameter of “trust-constr.” To ensure convergence to a proper distribution function, the sum of the probabilities was constrained to 1

$$\sum_{s}P_{\theta ,\lambda }(S=s|M)=1$$

In addition, the value of the probability of each sbin was constrained to a segment that strictly contain the [0,1] segment

$$-0.00005\leq P_{\theta ,\lambda }(S=s|M)\leq 1$$

When calculating the Kullback-Leibler divergences, the individual sbin probabilities were clamped to the [0.001, 1] segment. Following the convergence of the algorithm, any negative sbin probabilities were set to 0, and the probabilities were renormalized to ensure that they sum to 1.

As an initial validation for the cell positioning algorithm, we used all collected cells, effectively inferring the fraction of the cells residing in each of the spatial bins. Assuming that the mean cell size is similar across all bins, the area-normalized fraction provides an estimate of the thickness of the limb bud thickness at each spatial bin. As expected, the inferred limb bud thickness decreases as we move away from the embryo’s trunk and toward the edge of the limb bud.

### Inferring positions of metacells

The cells of a single metacell form a natural candidate for applying the cell positioning algorithm described in the previous section. We expect that the expression profiles of most of the limb bud’s cell types depend on spatial signaling fields. We can therefore assume that cells with a highly similar expression pattern (as the cells of a single metacell are guaranteed to be) are also tightly localized within the limb bud. The use of a metacell also provides a natural definition to the transcriptional criterion *M*, i.e., the inferred sbin distribution describes the expected spatial localization of new cells whose expression profile is close to the metacell’s centroid.

### Metacell-specific TFs

The metacell specificity of TFs was estimated using a method similar to the “roc” method in Seurat. For each gene, the empirical cumulative distribution function (CDF) was computed on the basis of the rescaled (from 0 to 1) gene expression in metacells. The area under the curve (AUC) was then computed. Genes that have very specific patterns of expression (i.e., expression in very few metacells) display large values of AUC because most metacells exhibit low values of gene expression.

### Chondrogenic trajectory and pseudo-time

Each chondrogenic progenitor was assigned to a compartment along the PD axis based on its color. Cells originating from other photoconversion patterns were assigned to the compartment for which their metacells had the highest probability. We performed principal components analysis on the top 2000 variable genes within each compartment (thus excluding variation resulting from spatial compartment-specific genes) and built graphs connecting each cell to its 40 nearest neighbors within its compartment. Projecting medial cells into the distal principal component (PC) space, we built a graph between distal and medial cells. Similarly, we built a graph connecting medial and proximal cells in the medial PC space. Edges were then filtered to keep at most 10 mutual edges. The resulting graph was projected using the DrL graph layout. ElPigraph (*77*) was then used to compute the pseudo-time for each cell. Gene expression as a function of the pseudo-time was computed and smoothed using loess regression.

## Supplementary Materials

### This PDF file includes:

Figs. S1 to S18 References
