# Supplementary material for: TATTOO-seq delineates spatial and cell type–specific regulatory programs in the developing limb
Source: Sci Adv. 2022 Dec 14;8(50):eadd0695. doi: 10.1126/sciadv.add0695 (PMC9750149; doi:10.1126/sciadv.add0695)
Supplement: Supplementary file 1 — Figs. S1 to S18 References [file sciadv.add0695_sm.pdf]

Science Advances
AAAS

Supplementary Materials for

TATTOO-seq delineates spatial and cell type–specific regulatory programs in the developing limb

Sébastien Bastide *et al.*

Corresponding author: François Spitz, fspitz@uchicago.edu

Sci. Adv. **8**, eadd0695 (2022)
DOI: 10.1126/sciadv.add0695

The PDF file includes:

Figs. S1 to S18
Legends for tables S1 to S5
References

Other Supplementary Material for this manuscript includes the following:

Tables S1 to S5

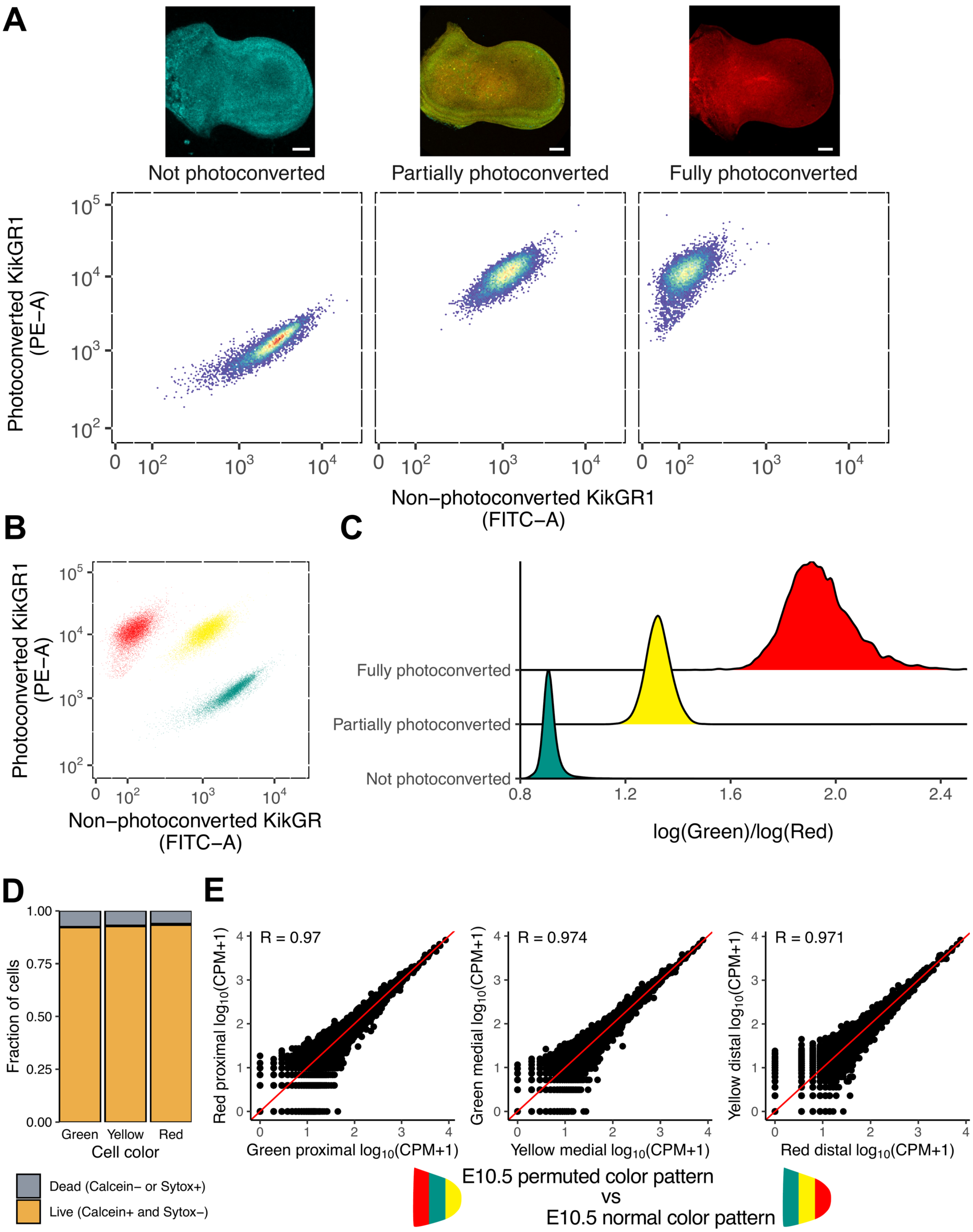

Fig. S1 Assessing the photoconversion efficiency and impact of the TATTOO-seq method on cell viability and transcriptome.

(A) Homogeneous distribution of green and red fluorescence in non-photoconverted, partially photoconverted and fully photoconverted whole limbs show the efficiency of photoconversion along the dorsal-ventral axis of the limb bud. Pictures of the corresponding limbs are shown above the flow cytometry plots. Scale bars = 100 µm. **(B)** Overlay of the three samples depicted in **(A)** show no overlap between the three colors. **(C)** Ridge plot of the ratio of the log-green and red fluorescence. **(D)** Fraction of live and dead cells (using Calcein and Sytox) assessed by flow cytometry for each color. The fraction of dead cells is independent from the color. **(E)** Comparison of aggregated gene expression for different degrees of photoconversion on the same spatial compartment in E10.5 wild-type mouse limbs processed with TATTOO-seq.

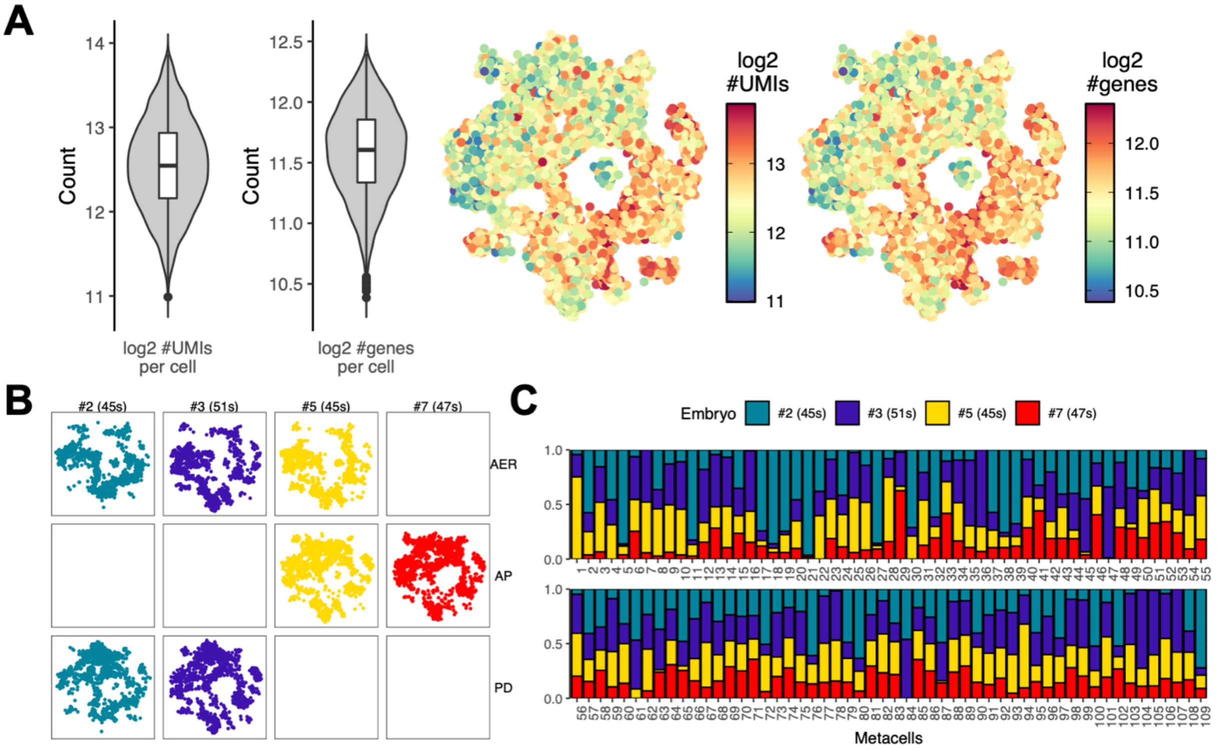

Fig. S2 Quality check for the TATTOO-seq data.

(A) Violin and 2D projection showing the distribution of the number of genes and number of UMIs detected per cell. The number of detected genes and UMIs is slightly higher in distal cells which are more actively proliferating. Median UMI count per cell = 5980, median number of detected genes = 3115. **(B)** 2D projection split by sample (columns) and photoconversion pattern (rows). **(C)** Bar chart showing the contribution of each embryo to each metacell.

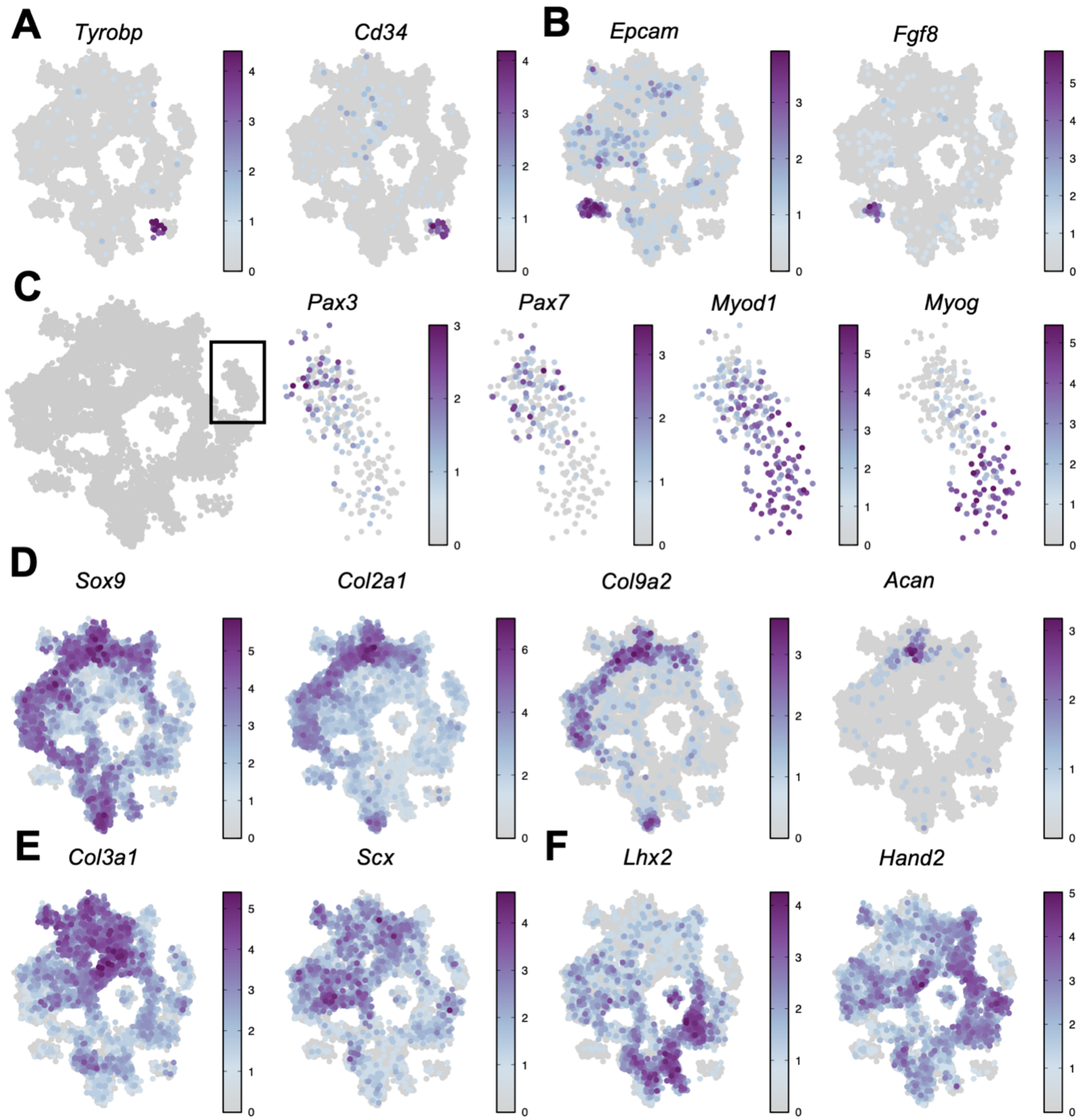

Fig. S3 Expression of cell type specific marker genes.

2D projection of the metacell graph. Cell color represents $\log_{10}$ depth-normalized counts for marker genes of **(A)** immune and endothelial cells, **(B)** epithelial cells (*Epcam*$^+$) are divided into two metacells: the *Fgf8*-expressing AER and the *Fgf8*-negative dorsal and ventral ectoderm, **(C)** three myogenic progenitor cell states corresponding to different stages of differentiation are detected, from *Pax3*-expressing progenitors migrating from the somites into the limb bud, to *Myod1*-expressing early differentiating myoblasts and *Myog*-expressing differentiated myocytes,

(D) chondrogenic progenitors, **(E)** dense regular connective tissue progenitors and **(F)** different populations of undifferentiated mesenchyme.

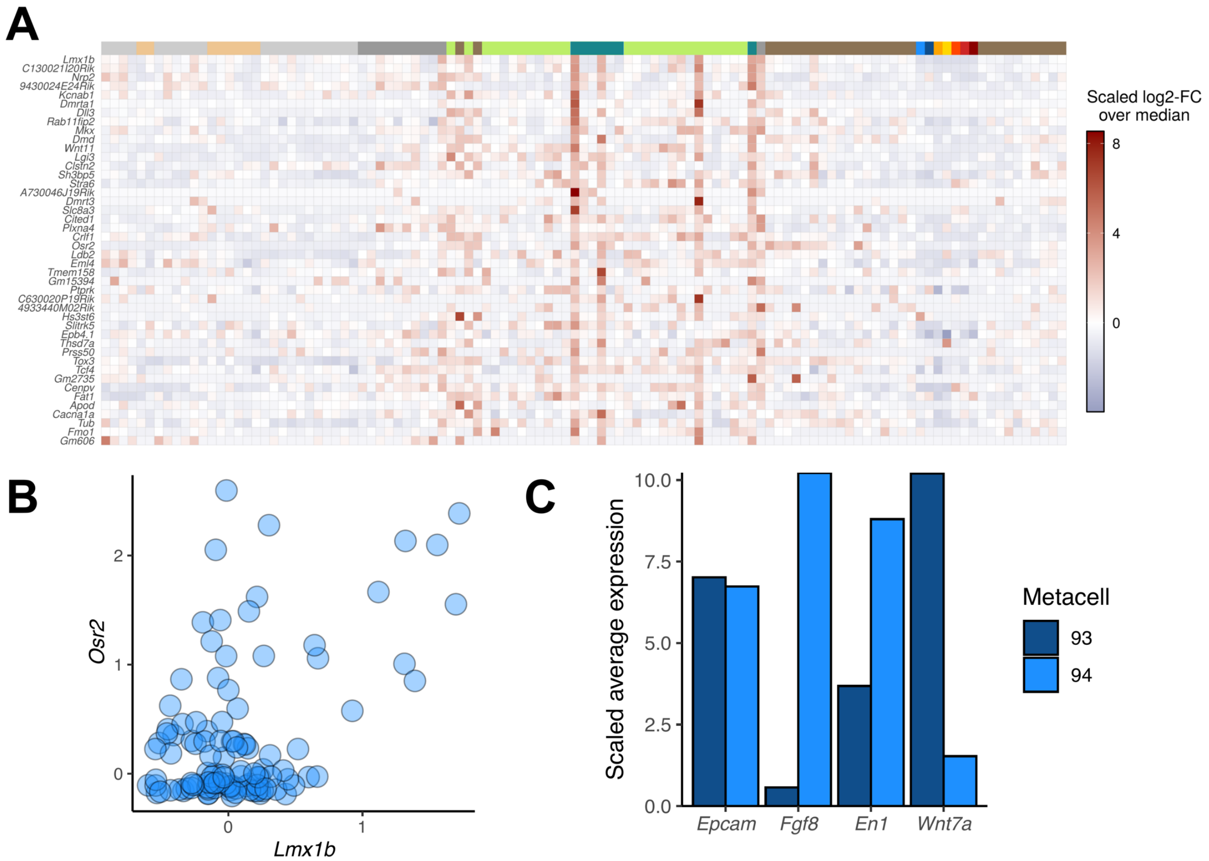

Fig. S4 DV organization of the limb cell states.

(A) *Lmx1b* was used as a marker of the dorsal mesenchyme (*81*). Heatmap showing the normalized expression of genes whose expression is loosely correlated or anti-correlated to that of *Lmx1b* (|R| > 0.4). **(B)** Scatter plot showing the expression of *Lmx1b* and *Osr2* for all metacells. Metacells exhibiting high levels of *Lmx1b* showed high levels of *Osr2*, a key regulator in connective tissue differentiation, suggesting that connective-tissue differentiation at this stage is preferentially occurring dorsally, as suggested by *Osr2 in situ* experiments (*82*). **(C)** Barplot of the expression of the AER marker *Fgf8*, the ventral marker *En1* and the dorsal marker *Wnt7a* in the *Epcam*$^{+}$ ectodermal metacells.

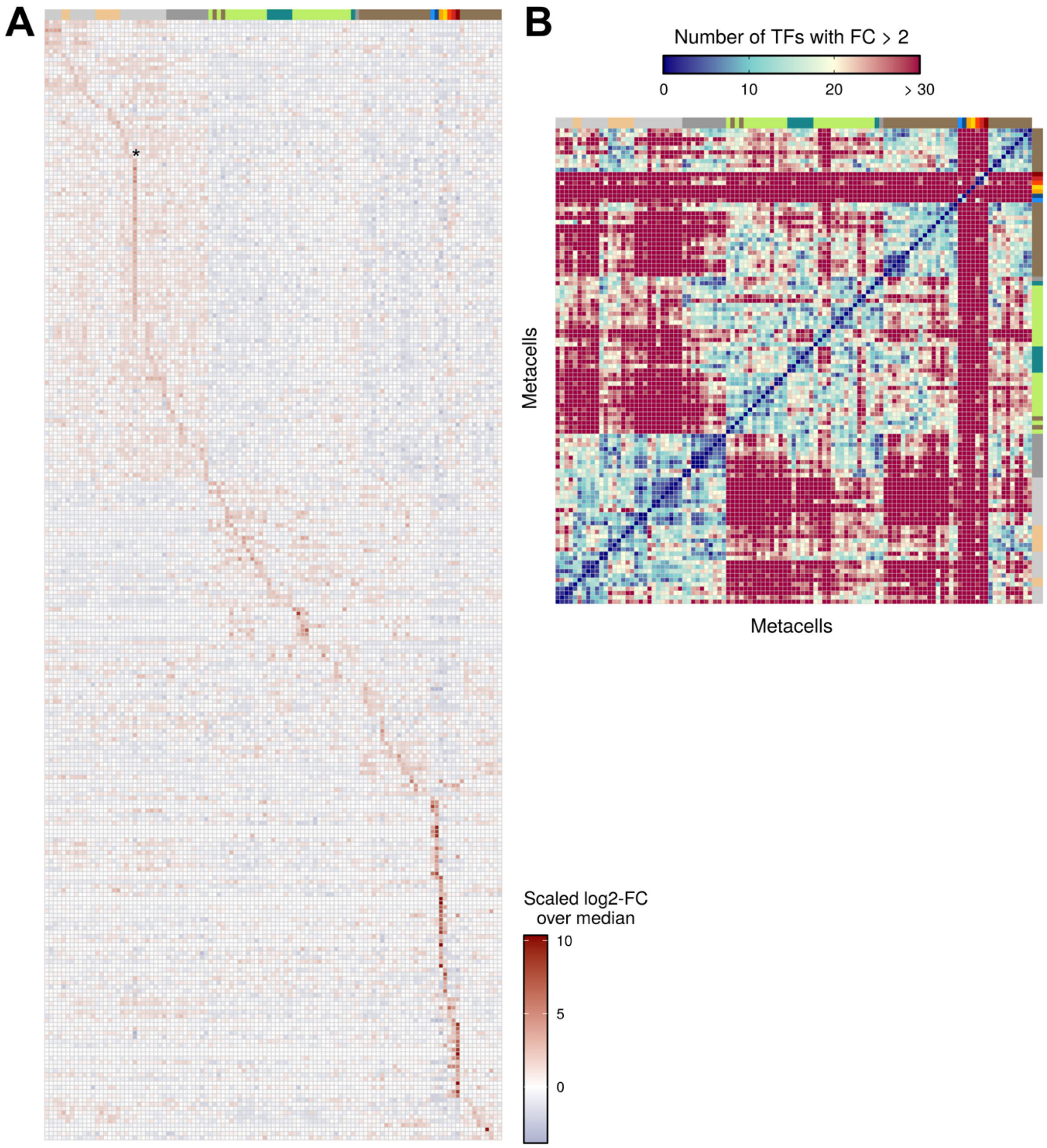

Fig. S5 Metacells exhibit a hierarchical and specific combinatorial code of TFs.

(A) Heatmap showing the normalized expression of genes with loosely specific gene expression (AUC > 0.5) across all metacells. * marks metacell 22 which groups cells of overall lower quality. **(B)** Heatmap showing the pairwise number of TFs that show different expression levels (FC > 2). Cell types are annotated using the same color code as in **Fig. 1**.

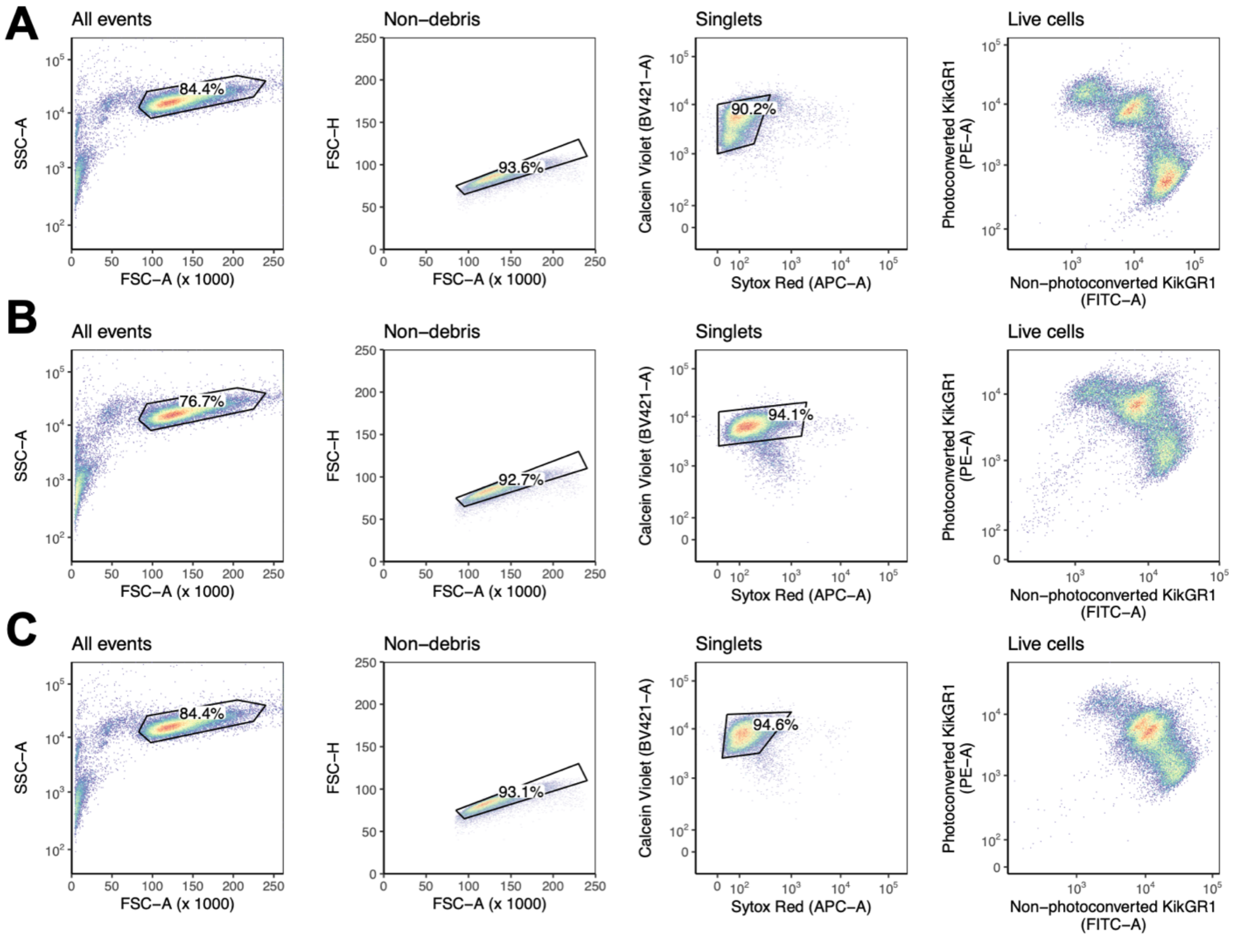

Fig. S6 Gating strategy for TATTOO-seq.

The gating strategy used for the PD **(A)**, AP **(B)** and AER **(C)** photoconversion patterns. Debris are first filtered out using SSC-A vs FSC-A, then singlets are recovered by looking at FSC-H vs FSC-A. Finally, live cells are recovered by inspecting Calcein Violet vs Sytox Red. Non-green and non-red cells are then excluded. No further gating on Red vs Green is performed.

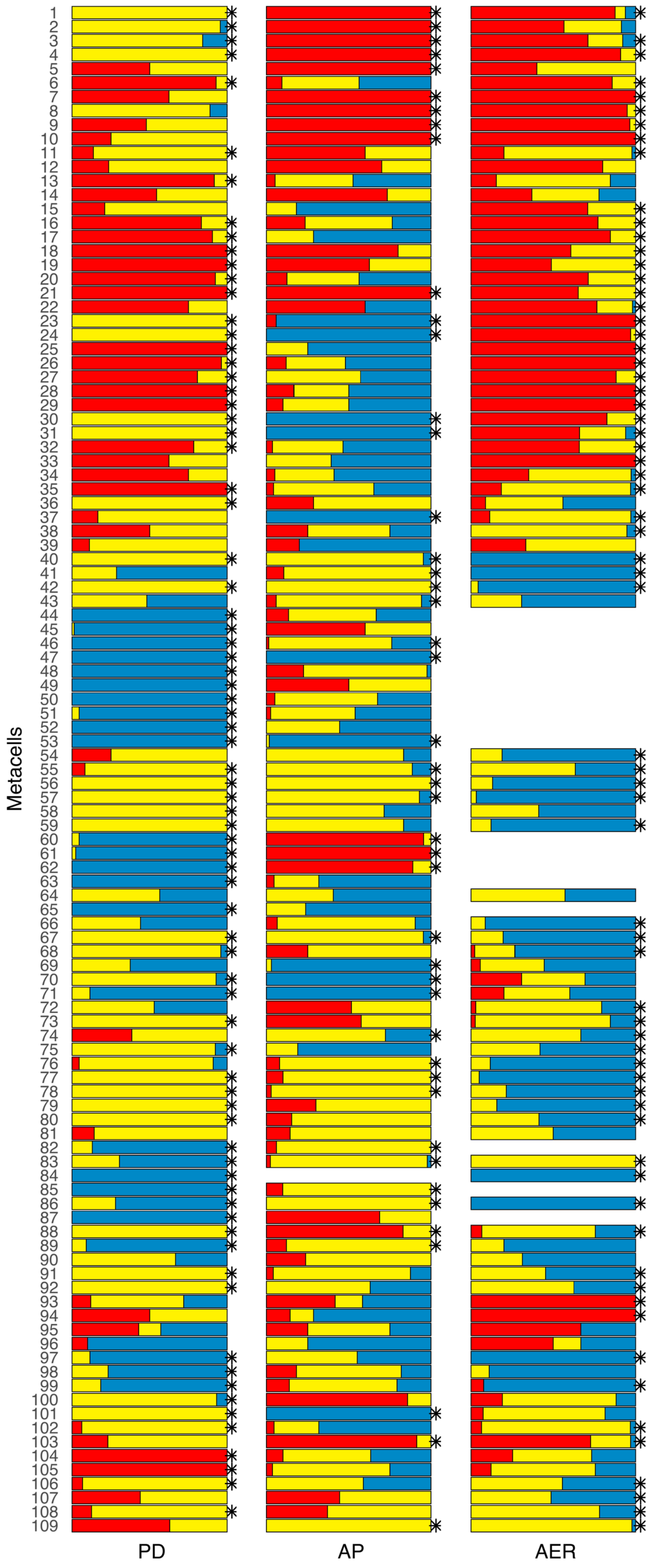
Metacells
PD
AP
AER

Fig. S7 Metacells are spatially homogeneous.

We employed Shannon entropy as a metric for measuring color heterogeneity. Monte Carlo simulations ($n = 100000$) were used to test for positional homogeneity. Stacked bar plots showing the distribution of color for each metacell in all three photoconversion patterns (PD, AP and AER). Clusters labeled with a start (*) display significantly lower Shannon entropy than expected by chance (Bonferroni corrected p-value ≤ 0.01).

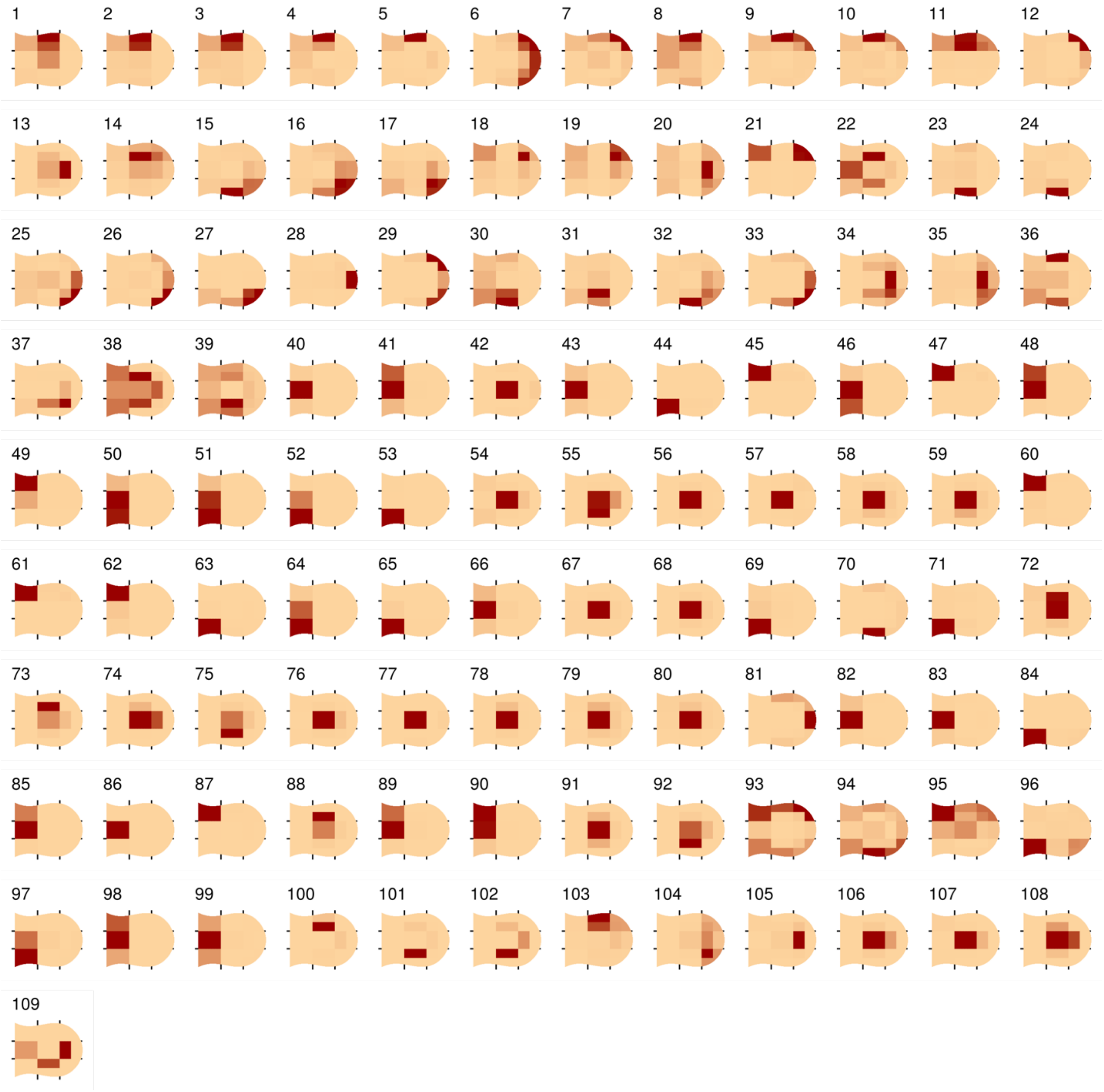

Fig. S8 Mouse limb bud metacell positions.

Spatial distribution for all mouse limb bud metacells.

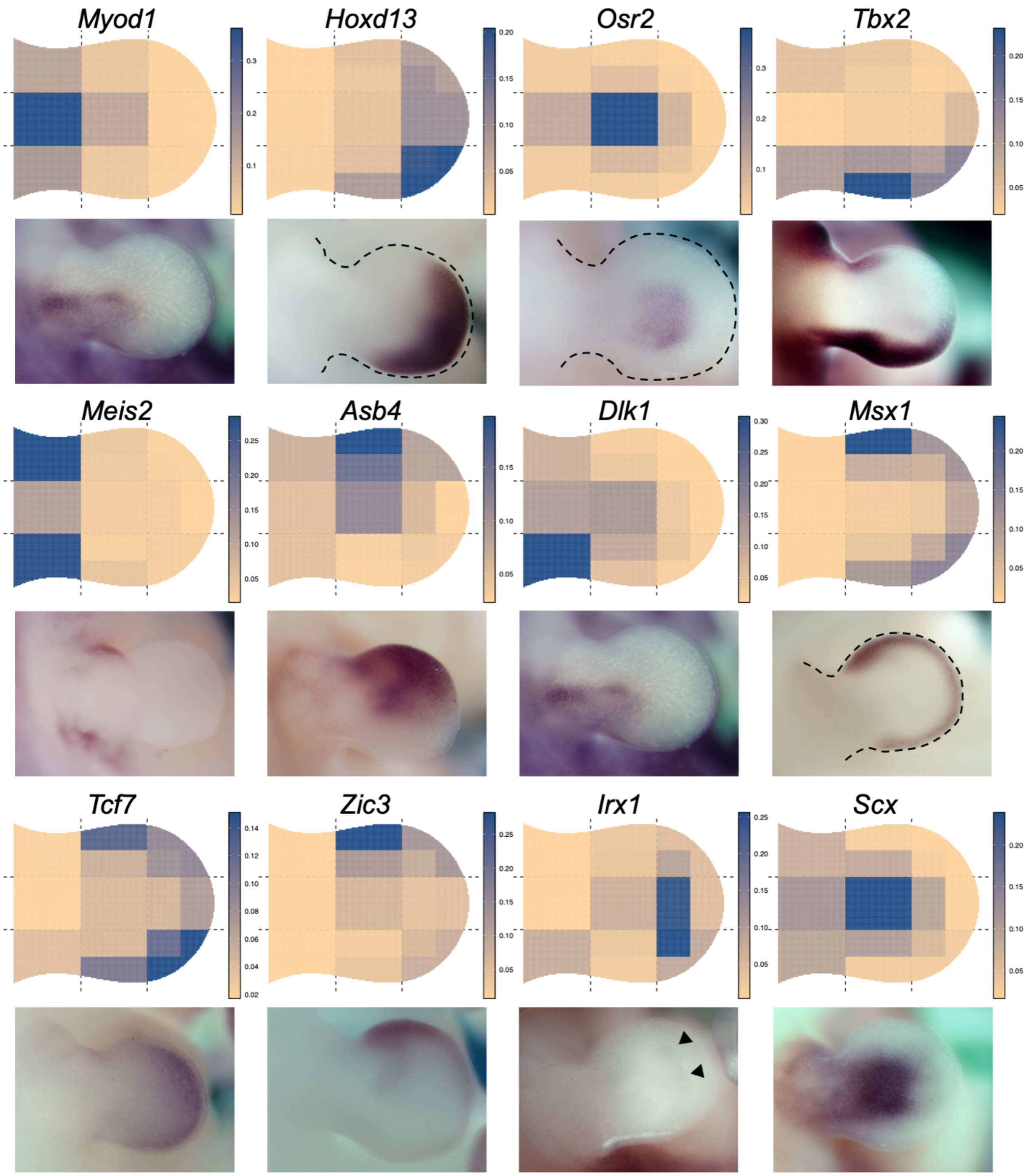

Fig. S9 vISH match the gene expression patterns obtained by classical *in situ* hybridization. vISH and the corresponding classical ISH patterns for twelve TFs involved in limb development. ISH images were obtained from the EMBRYS database (*79*).

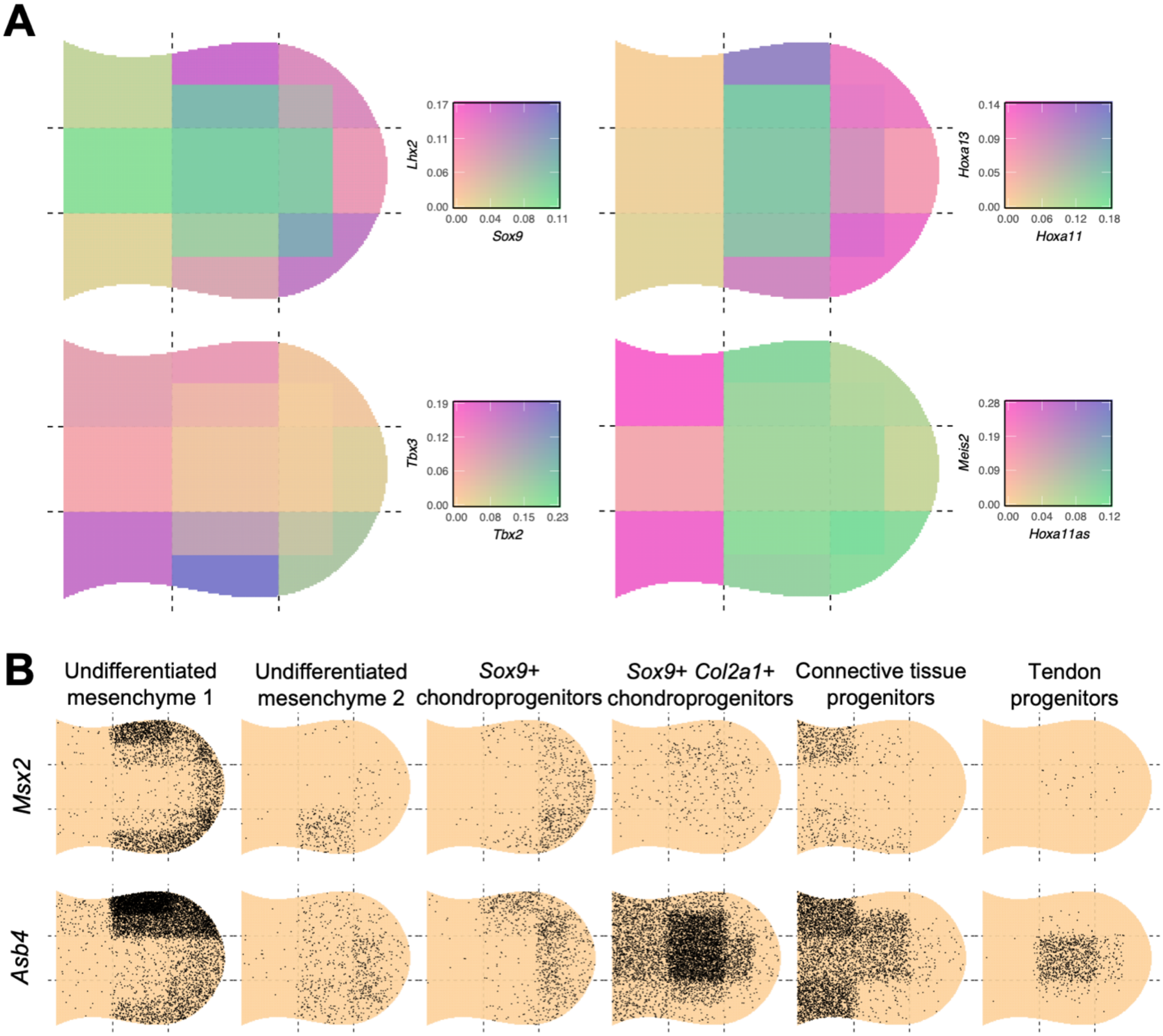

Fig. S10 Double virtual *in situ* hybridization (vISH) and cell type-specific vISH.

(A) Double vISH uses a bivariate color scale to represent the spatial overlap of expression patterns.

(B) vISH can be deconvolved into cell type-specific vISH. To take into account the different dynamic ranges across cell types, up to 10,000 UMIs are sampled according to the expected number of counts for each spatial bin.

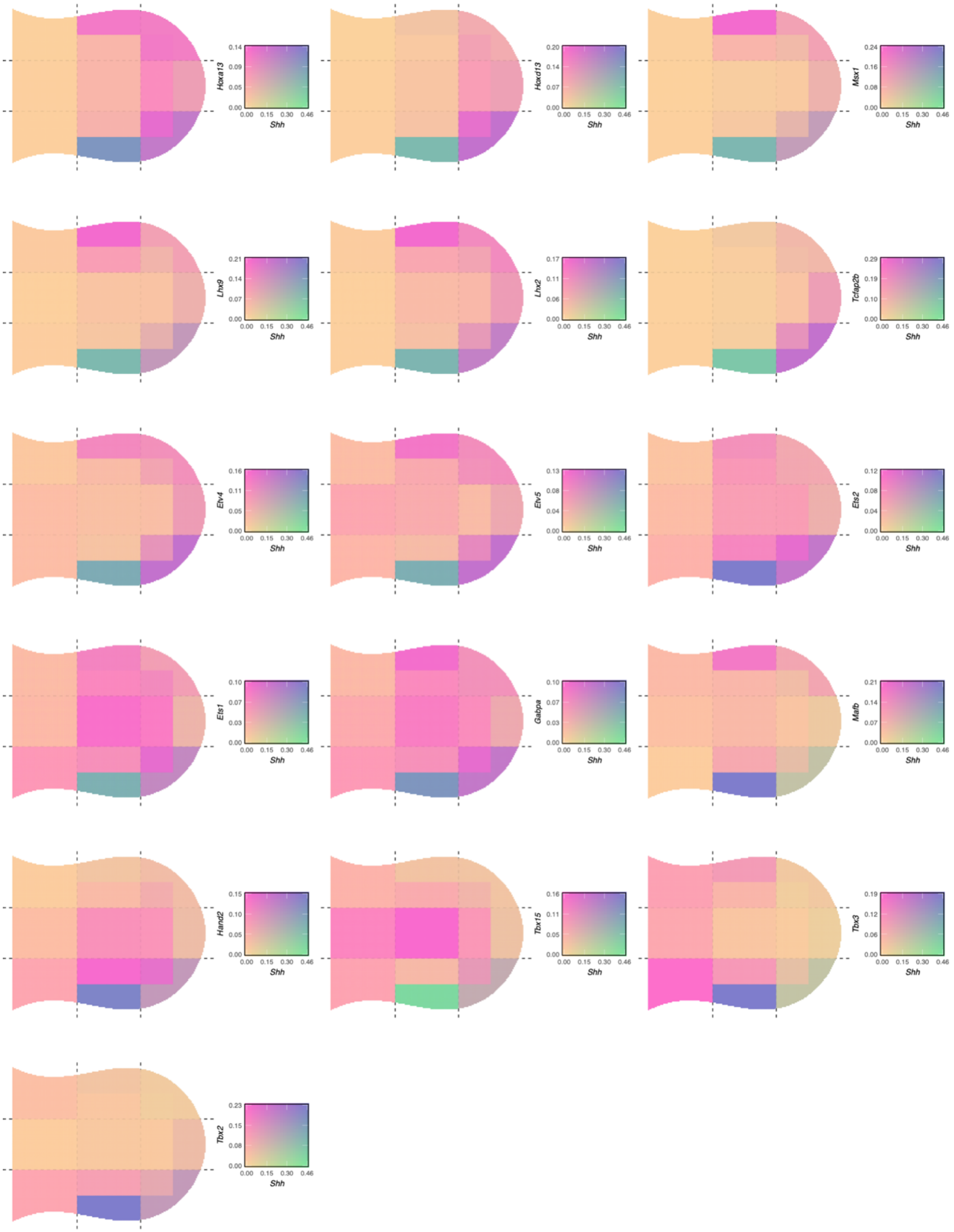

Fig. S11 Spatial domains of expression of potential regulators of *Shh* expression in the ZPA. Double vISH for Shh and transcription factors shown in **Fig. 2**.

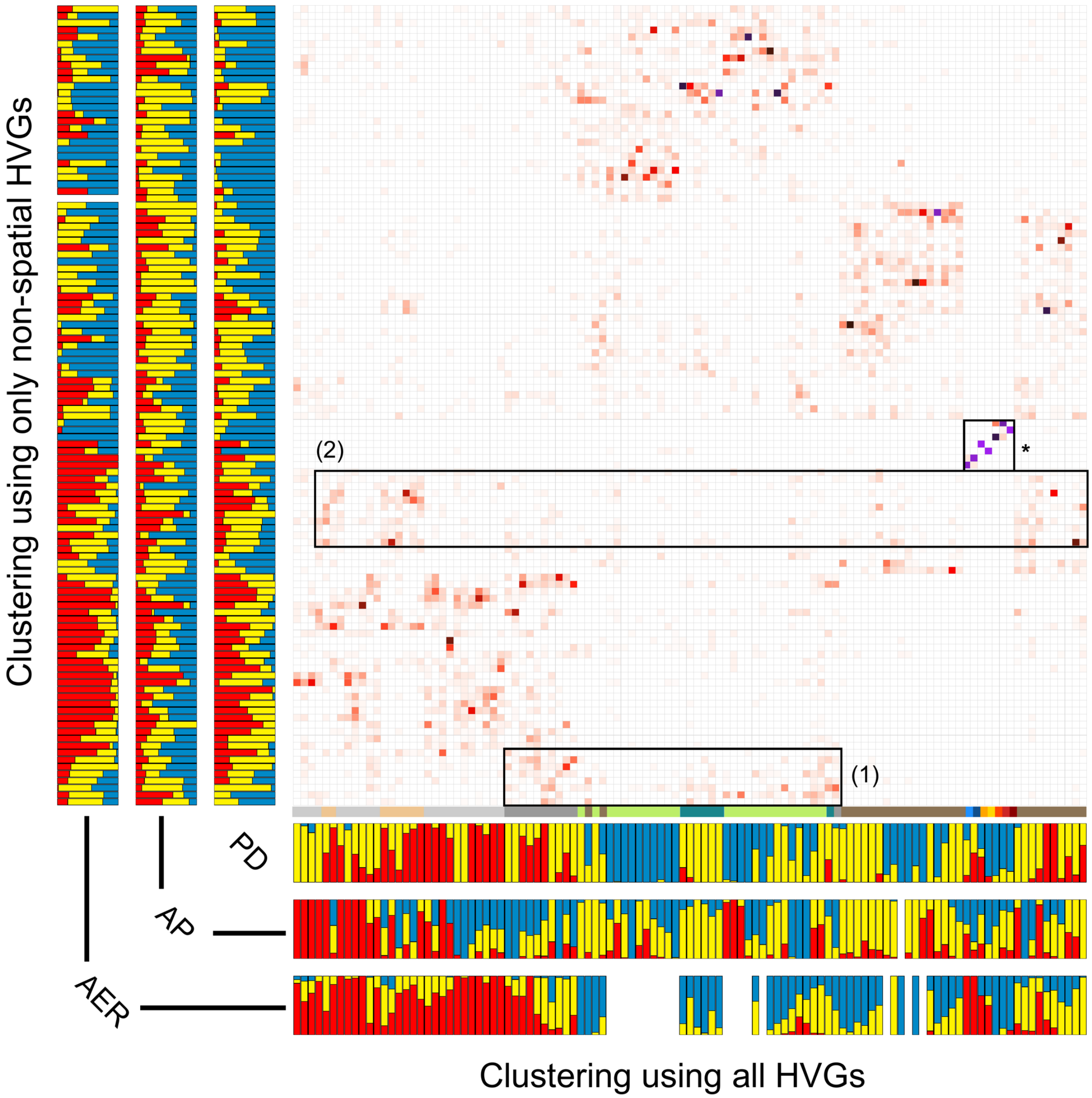

Fig. S12 Confusion matrix between the two dataset clusterings.

Confusion matrix representing the redistribution of cells between the clustering using all the highly variable genes and the clustering using only non-spatial highly variable genes. Darker values (black, purple) indicate high correspondence between metacells of the two clusterings. The color distribution is indicated on the side of the heatmap. Non-mesenchymal clusters are unaffected (*) while some mesenchymal clusters are split and merged. Some no-HVGs metacells clusters receive contribution from type 2 undifferentiated mesenchyme and dense regular connective tissue progenitors metacells (1), and from distal chondrogenic progenitors and proximal *Sox9*$^{+}$ *Col2a1*$^{+}$

chondroprogenitors (2). Blacklisting of spatial HVGs additionally results in extensive cell type internal reorganization. Abrogating spatial information potentially leads to artefactual clustering if cell types are homogeneous.

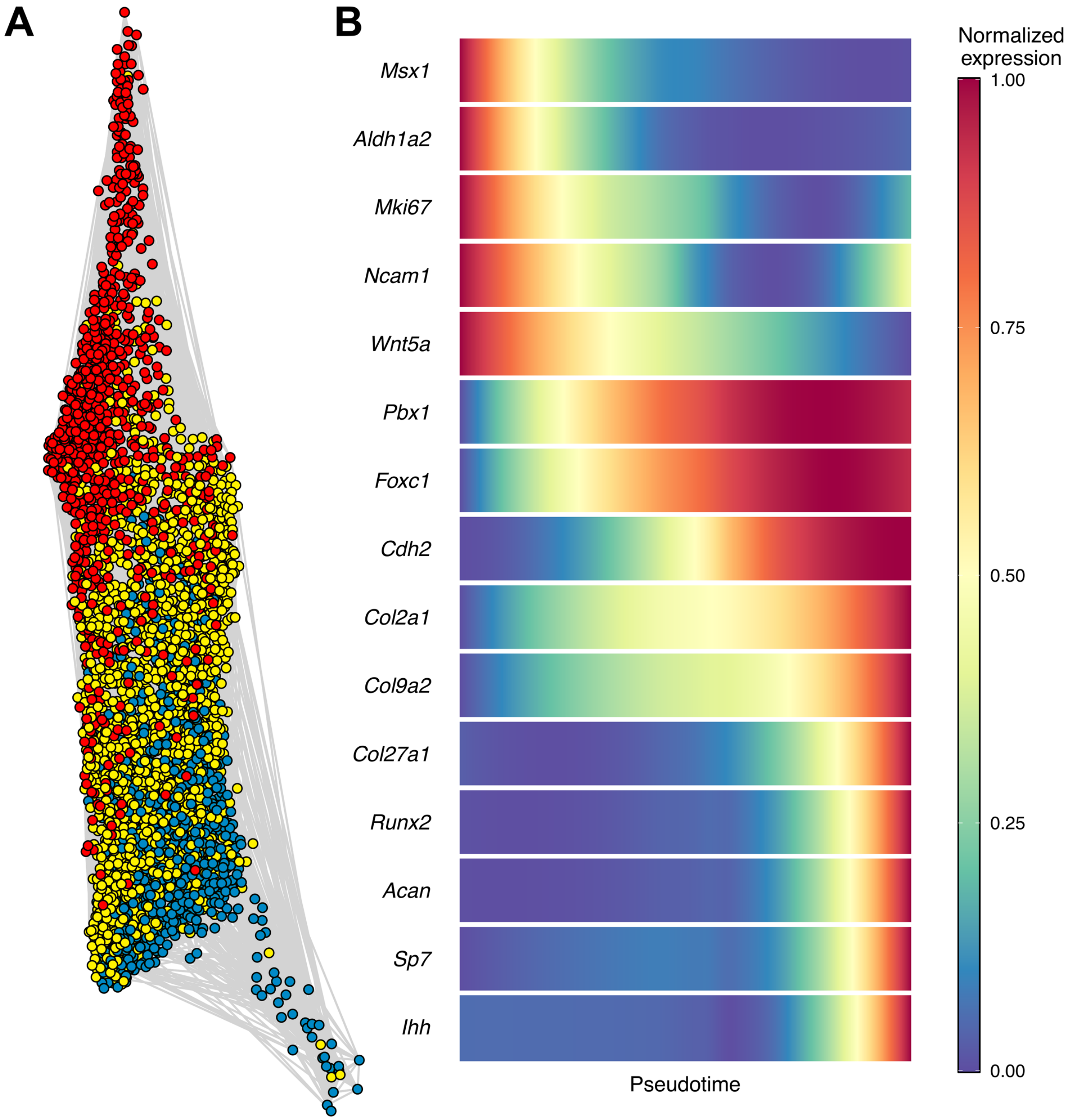

Fig. S13 Eliminating confounder effects of cell position reveals overlapping chondrogenic transcriptomic states.

(A) 2D projection of the k-NN (k = 10) graph of chondrocytes using the force-directed Distributed Recursive (Graph) Layout algorithm. The procedure used to generate the graph is described in the **methods** section. **(B)** Pseudotime was assessed using ElPiGraph (*77*) and gene expression as a function of pseudotime was estimated and smoothed using a loess regression for various stage-specific chondrogenesis markers.

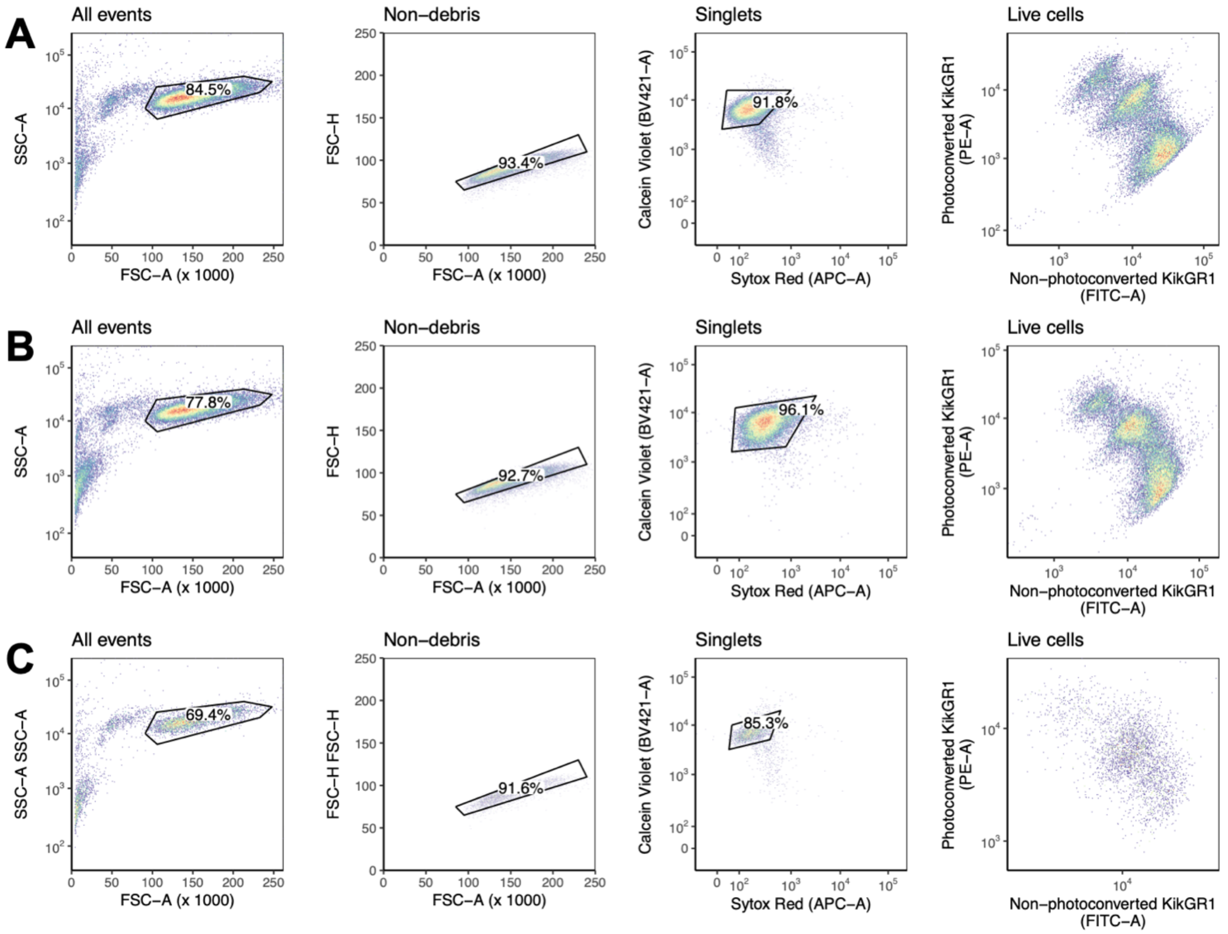

Fig. S14 Gating strategy for TATTOO-seq on mutant embryos.

The gating strategy used for the PD **(A)**, AP **(B)** and AER **(C)** photoconversion patterns. Debris are first filtered out using SSC-A vs FSC-A, then singlets are recovered by looking at FSC-H vs FSC-A. Finally, live cells are recovered by inspecting Calcein Violet vs Sytox Red. Non-green and non-red cells are then excluded. No further gating on Red vs Green is performed.

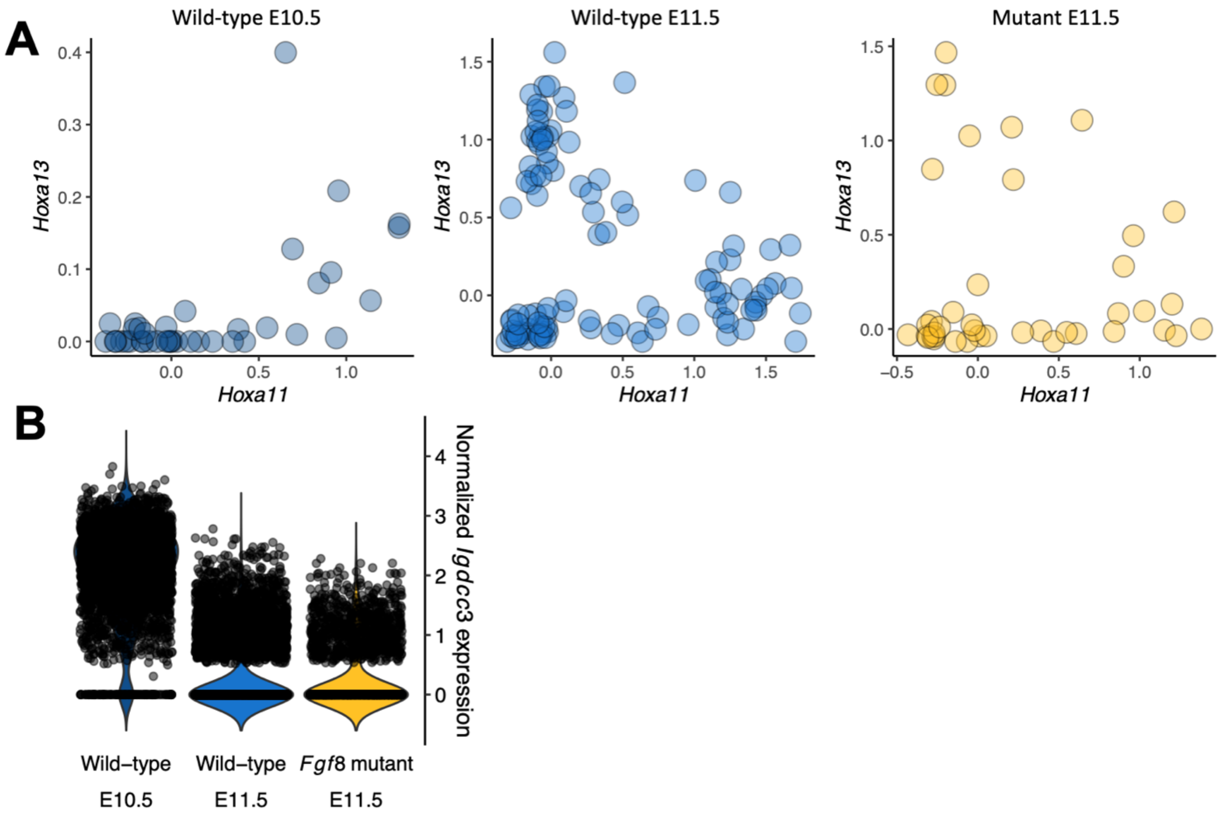

Fig. S15 Differences between wild-type and *Fgf8* mutant cells do not reflect a global developmental delay in mutant limbs.

(A) Scatter plot showing the normalized expression of *Hoxa13* and *Hoxa11* in E10.5 and E11.5 wild-type metacells and E11.5 mutant metacells. In the mutant limbs, *Hoxa13* and *Hoxa11* were expressed in distinct cell-states, corresponding to the largely mutually exclusive territories observed in wild-type E11.5 limbs, and contrasting with the nested expression found earlier at E10.5 (*78*, *83*). **(B)** Violin plot showing the $\log_{10}$ depth-normalized expression of *Igdcc3* in E10.5 and E11.5 wild-type cells and E11.5 mutant cells. *Igdcc3* has been reported to be a sensitive temporal marker of limb development, showing strong expression at E10.5 but minimal expression at E11.5 (*84*). We found its expression greatly reduced in both wild-type and mutant E11.5 scRNA-seq data, while it is robustly detected in E10.5 samples.

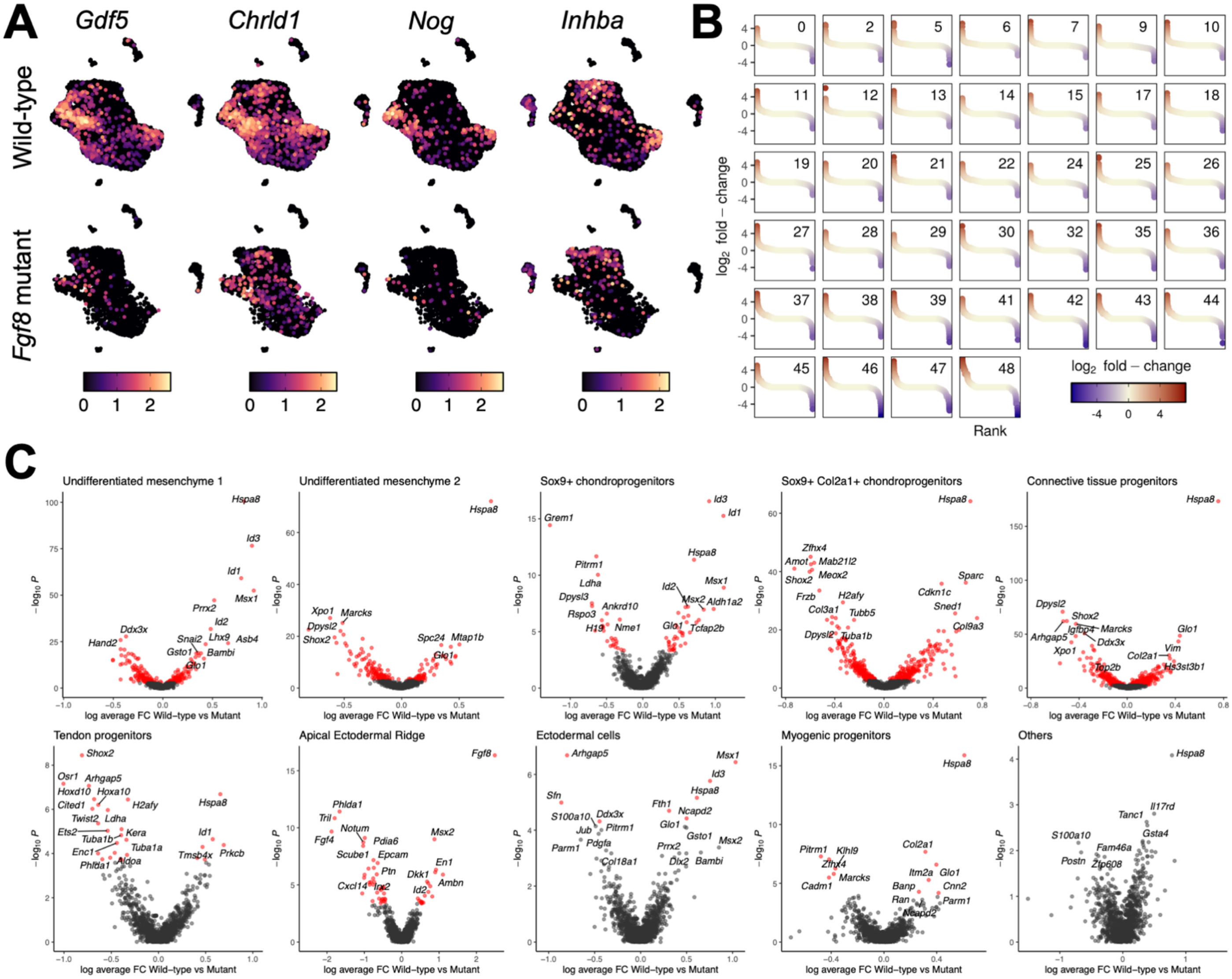

Fig. S16 Transcriptional differences between wild-type and mutant limb cells.

(A) UMAP showing $\log_{10}$ depth-normalized counts for some genes associated with the TGFβ superfamily signaling showing a reduction of expression in mutant cells. The data is split by genotype: wild-type (top row) and *Fgf8* mutant (bottom row). **(B)** Plot showing the distribution of $\log_2$ fold-changes between wild-type and mutant cells within each Seurat cluster. **(C)** Volcano plot showing the p-values of statistical tests for differential expression as a function of $\log_2$ fold-change between wild-type and mutant cells in each cell type. Genes for which BH-corrected p-value < 0.01 are represented in red and genes names are indicated for the top 20 most significant genes.

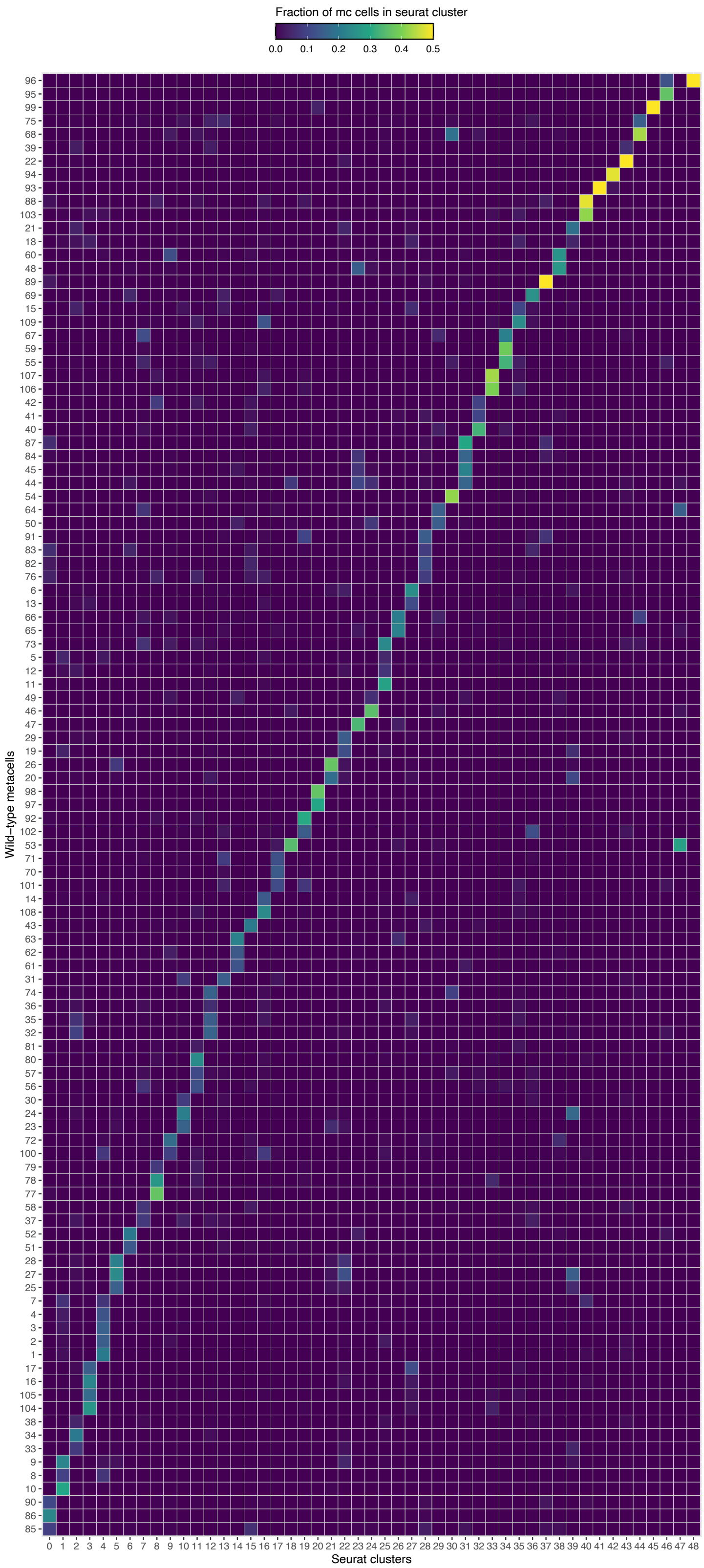

Figure S17 Correspondence between wild-type metacells and Seurat clusters

Contingency table describing the relationship between wild-type metacells and Seurat clusters. Each cell shows the fraction of cell from a given metacell in each Seurat cluster.

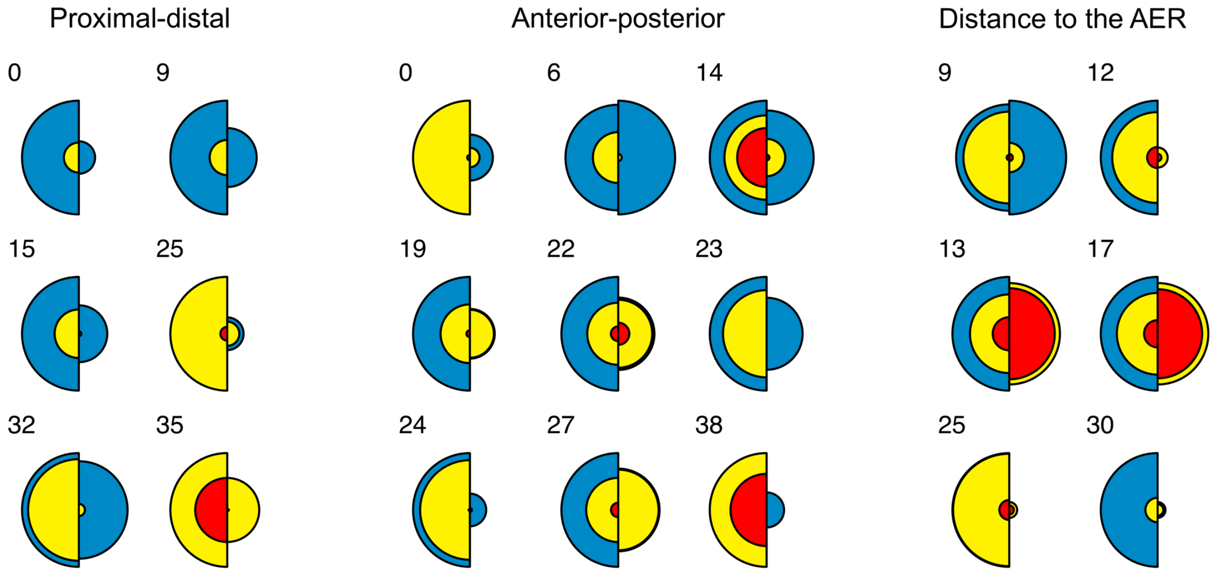

Fig. S18 Spatial distribution changes between wild-type and mutant limb cells.

Circular histograms representing the distribution of colors for wild-type and mutant cells in Seurat clusters that show altered spatial distributions.

Other Supplementary Materials for this manuscript include the following:

Table S1. Gene expression in the metacells of the wild-type forelimb bud
Table S2. "Spatially regulated" genes in the forelimb bud
Table S3. Differentially accessible peaks in the *Sox9* regulatory domain
Table S4. Gene expression in the Seurat clusters comprising wild-type and DEL(PolL-SHFM) forelimb cells
Table S5. Cell type-specific differential expression in DEL(PolL-SHFM) mutant forelimbs

REFERENCES AND NOTES

1. L. Wolpert, Positional information and the spatial pattern of cellular differentiation. *J. Theor. Biol.* **25**, 1–47 (1969).

2. F. R. Goodman, Congenital abnormalities of body patterning: Embryology revisited. *Lancet* **362**, 651–662 (2003).

3. L. A. Lettice, A. E. Hill, P. S. Devenney, R. E. Hill, Point mutations in a distant sonic hedgehog cis-regulator generate a variable regulatory output responsible for preaxial polydactyly. *Hum. Mol. Genet.* **17**, 978–985 (2008).

4. E. Z. Kvon, O. K. Kamneva, U. S. Melo, I. Barozzi, M. Osterwalder, B. J. Mannion, V. Tissières, C. S. Pickle, I. Plajzer-Frick, E. A. Lee, M. Kato, T. H. Garvin, J. A. Akiyama, V. Afzal, J. Lopez-Rios, E. M. Rubin, D. E. Dickel, L. A. Pennacchio, A. Visel, Progressive loss of function in a limb enhancer during snake evolution. *Cell* **167**, 633–642.e11 (2016).

5. M. Towers, Evolution of antero-posterior patterning of the limb: Insights from the chick. *Genesis* **56**, e23047 (2018).

6. A. Abzhanov, M. Protas, B. R. Grant, P. R. Grant, C. J. Tabin, Bmp4 and morphological variation of beaks in Darwin's finches. *Science* **305**, 1462–1465 (2004).

7. A. Zuniga, Next generation limb development and evolution: Old questions, new perspectives. *Development* **142**, 3810–3820 (2015).

8. J. Cao, M. Spielmann, X. Qiu, X. Huang, D. M. Ibrahim, A. J. Hill, F. Zhang, S. Mundlos, L. Christiansen, F. J. Steemers, C. Trapnell, J. Shendure, The single-cell transcriptional landscape of mammalian organogenesis. *Nature* **566**, 496–502 (2019).

9. R. Satija, J. A. Farrell, D. Gennert, A. F. Schier, A. Regev, Spatial reconstruction of single-cell gene expression data. *Nat. Biotechnol.* **33**, 495–502 (2015).

10. K. Achim, J.-B. Pettit, L. R. Saraiva, D. Gavriouchkina, T. Larsson, D. Arendt, J. C. Marioni, High-throughput spatial mapping of single-cell RNA-seq data to tissue of origin. *Nat.*

Biotechnol. **33**, 503–509 (2015).

11. E. Lubeck, A. F. Coskun, T. Zhiyentayev, M. Ahmad, L. Cai, Single-cell in situ RNA profiling by sequential hybridization. *Nat. Methods* **11** 360–361 (2014).

12. K. H. Chen, A. N. Boettiger, J. R. Moffitt, S. Wang, X. Zhuang, Spatially resolved, highly multiplexed RNA profiling in single cells. *Science* **348**, aaa609 (2015).

13. J. H. Lee, E. R. Daugharthy, J. Scheiman, R. Kalhor, T. C. Ferrante, R. Terry, B. M. Turczyk, J. L. Yang, H. S. Lee, J. Aach, K. Zhang, G. M. Church, Fluorescent in situ sequencing (FISSEQ) of RNA for gene expression profiling in intact cells and tissues. *Nat. Protoc.* **10**, 442–458 (2015).

14. X. Wang, W. E. Allen, M. A. Wright, E. L. Sylwestrak, N. Samusik, S. Vesuna, K. Evans, C. Liu, C. Ramakrishnan, J. Liu, G. P. Nolan, F.-A. Bava, K. Deisseroth, Three-dimensional intact-tissue sequencing of single-cell transcriptional states. *Science* **361**, eaat5691 (2018).

15. S. G. Rodriques, R. R. Stickels, A. Goeva, C. A. Martin, E. Murray, C. R. Vanderburg, J. Welch, L. M. Chen, F. Chen, E. Z. Macosko, Slide-seq: A scalable technology for measuring genome-wide expression at high spatial resolution. *Science* **363**, 1463–1467 (2019).

16. A. Chen, S. Liao, M. Cheng, K. Ma, L. Wu, Y. Lai, X. Qiu, J. Yang, J. Xu, S. Hao, X. Wang, H. Lu, X. Chen, X. Liu, X. Huang, Z. Li, Y. Hong, Y. Jiang, J. Peng, S. Liu, M. Shen, C. Liu, Q. Li, Y. Yuan, X. Wei, H. Zheng, W. Feng, Z. Wang, Y. Liu, Z. Wang, Y. Yang, H. Xiang, L. Han, B. Qin, P. Guo, G. Lai, P. Muñoz-Cánoves, P. H. Maxwell, J. P. Thiery, Q.-F. Wu, F. Zhao, B. Chen, M. Li, X. Dai, S. Wang, H. Kuang, J. Hui, L. Wang, J.-F. Fei, O. Wang, X. Wei, H. Lu, B. Wang, S. Liu, Y. Gu, M. Ni, W. Zhang, F. Mu, Y. Yin, H. Yang, M. Lisby, R. J. Cornall, J. Mulder, M. Uhlén, M. A. Esteban, Y. Li, L. Liu, X. Xu, J. Wang, Spatiotemporal transcriptomic atlas of mouse organogenesis using DNA nanoball-patterned arrays. *Cell* **185**:1777–1792.e21 (2022).

17. C. Tabin, L. Wolpert, Rethinking the proximodistal axis of the vertebrate limb in the molecular era. *Genes Dev.* **21**, 1433–1442 (2007).

18. H. Shimizu, S. Yokoyama, H. Asahara, Growth and differentiation of the developing limb bud from the perspective of chondrogenesis. *Dev. Growth Differ.* **49** , 449–454 (2007).

19. M. Buckingham, P. W. J. Rigby, Gene regulatory networks and transcriptional mechanisms that control myogenesis. *Dev. Cell* **28**, 225–238 (2014).

20. S. Nassari, D. Duprez, C. Fournier-Thibault, Non-myogenic contribution to muscle development and homeostasis: The role of connective tissues. *Front. Cell Dev. Biol.* **5**, 22 (2017).

21. K. L. Cooper, K. E. Sears, A. Uygur, J. Maier, K.-S. Baczkowski, M. Brosnahan, D. Antczak, J. A. Skidmore, C. J. Tabin, Patterning and post-patterning modes of evolutionary digit loss in mammals. *Nature* **511**, 41–45 (2014).

22. D. Basel, A. DePaepe, M. Kilpatrick, P. Tsipouras, Split hand foot malformation is associated with a reduced level of Dactylin gene expression. *Clin. Genet.* **64**, 350–354 (2003).

23. L. A. Lettice, S. J. H. Heaney, L. A. Purdie, L. Li, P. de Beer, B. A. Oostra, D. Goode, G. Elgar, R. E. Hill, E. de Graaff, A long-range Shh enhancer regulates expression in the developing limb and fin and is associated with preaxial polydactyly. *Hum. Mol. Genet.* **12**, 1725–1735 (2003).

24. J. Kohlhase, A. Wischermann, H. Reichenbach, U. Froster, W. Engel, Mutations in the SALL1 putative transcription factor gene cause Townes-Brocks syndrome. *Nat. Genet.* **18**, 81–83 (1998).

25. P. Hill, B. Wang, U. Rüther, The molecular basis of Pallister Hall associated polydactyly. *Hum. Mol. Genet.* **16**, 2089–2096 (2007).

26. R. D. Riddle, R. L. Johnson, E. Laufer, C. Tabin, Sonic hedgehog mediates the polarizing activity of the ZPA. *Cell* **75**, 1401–1416 (1993).

27. M. Lewandoski, X. Sun, G. R. Martin, Fgf8 signalling from the AER is essential for normal

limb development. *Nat. Genet.* **26**, 460–463 (2000).

28. H. Akiyama, J.-E. Kim, K. Nakashima, G. Balmes, N. Iwai, J. M. Deng, Z. Zhang, J. F. Martin, R. R. Behringer, T. Nakamura, B. De Crombrugghe, Osteo-chondroprogenitor cells are derived from Sox9 expressing precursors. *Proc. Natl. Acad. Sci. U.S.A.* **102**, 14665–14670 (2005).

29. Y. J. Wang, R. M. Belflower, Y.-F. Dong, E. M. Schwarz, R. J. O'Keefe, H. Drissi, Runx1/AML1/Cbfa2 mediates onset of mesenchymal cell differentiation toward chondrogenesis. *J. Bone Miner. Res.* **20**, 1624–1636 (2005).

30. I. Tzchori, T. F. Day, P. J. Carolan, Y. Zhao, C. A. Wassif, L. Q. Li, M. Lewandoski, M. Gorivodsky, P. E. Love, F. D. Porter, H. Westphal, Y. Yang, LIM homeobox transcription factors integrate signaling events that control three-dimensional limb patterning and growth. *Development* **136**, 1375–1385 (2009).

31. S. Yokoyama, S. Furukawa, S. Kitada, M. Mori, T. Saito, K. Kawakami, J. C. I. Belmonte, Y. Kawakami, Y. Ito, T. Sato, H. Asahara, Analysis of transcription factors expressed at the anterior mouse limb bud. *PLOS ONE* **12**, e0175673 (2017).

32. S. Nowotschin, A.-K. Hadjantonakis, Use of KikGR a photoconvertible green-to-red fluorescent protein for cell labeling and lineage analysis in ES cells and mouse embryos. *BMC Dev. Biol.* **9**, 49 (2009).

33. D. A. Jaitin, E. Kenigsberg, H. Keren-Shaul, N. Elefant, F. Paul, I. Zaretsky, A. Mildner, N. Cohen, S. Jung, A. Tanay, I. Amit, Massively parallel single-cell RNA-seq for marker-free decomposition of tissues into cell types. *Science* **343**, 776–779 (2014).

34. C. Medaglia, A. Giladi, L. Stoler-Barak, M. De Giovanni, T. M. Salame, A. Biram, E. David, H. Li, M. Iannacone, Z. Shulman, I. Amit, Spatial reconstruction of immune niches by combining photoactivatable reporters and scRNA-seq. *Science* **358**, 1622–1626 (2017).

35. Y. Baran, A. Bercovich, A. Sebe-Pedros, Y. Lubling, A. Giladi, E. Chomsky, Z. Meir, M. Hoichman, A. Lifshitz, A. Tanay, MetaCell: Analysis of single-cell RNA-seq data using K-

nn graph partitions. *Genome Biol.* **20**, 206 (2019).

36. C. A. Loomis, R. A. Kimmel, C. X. Tong, J. Michaud, A. L. Joyner, Analysis of the genetic pathway leading to formation of ectopic apical ectodermal ridges in mouse Engrailed-1 mutant limbs. *Development* **125**, 1137–1148 (1998).

37. L. A. Lettice, I. Williamson, J. H. Wiltshire, S. Peluso, P. S. Devenney, A. E. Hill, A. Essafi, J. Hagman, R. Mort, G. Grimes, C. L. DeAngelis, R. E. Hill, Opposing functions of the ETS factor family define *Shh* spatial expression in limb buds and underlie polydactyly. *Dev. Cell* **22**, 459–467 (2012).

38. M. Osterwalder, D. Speziale, M. Shoukry, R. Mohan, R. Ivanek, M. Kohler, C. Beisel, X. Wen, S. J. Scales, V. M. Christoffels, A. Visel, J. Lopez-Rios, R. Zeller, HAND2 targets define a network of transcriptional regulators that compartmentalize the early limb bud mesenchyme. *Dev. Cell* **31**, 345–357 (2014).

39. L. A. Lettice, P. Devenney, C. De Angelis, R. E. Hill, The conserved sonic hedgehog limb enhancer consists of discrete functional elements that regulate precise spatial expression. *Cell Rep.* **20**, 1396–1408 (2017).

40. T. Sagai, M. Hosoya, Y. Mizushina, M. Tamura, T. Shiroishi, Elimination of a long-range cis-regulatory module causes complete loss of limb-specific Shh expression and truncation of the mouse limb. *Development* **132**, 797–803 (2005).

41. C. A. Gurnett, A. M. Bowcock, F. R. Dietz, J. A. Morcuende, J. C. Murray, M. B. Dobbs, Two novel point mutations in the long-range SHH enhancer in three families with triphalangeal thumb and preaxial polydactyly. *Am. J. Med. Genet. Part A.* **143A**, 27–32 (2007).

42. D. Wieczorek, B. Pawlik, Y. Li, N. A. Akarsu, A. Caliebe, K. J. W. May, B. Schweiger, F. R. Vargas, S. Balci, G. Gillessen-Kaesbach, B. Wollnik, A specific mutation in the distant sonic hedgehog (*SHH*) cis-regulator (ZRS) causes Werner mesomelic syndrome (WMS) while complete ZRS duplications underlie Haas type polysyndactyly and preaxial polydactyly

(PPD) with or without triphalangeal thumb. *Hum. Mutat.* **31**, 81–89 (2010).

43. C. Xu, X. Yang, H. Zhou, Y. Li, C. Xing, T. Zhou, D. Zhong, C. Lian, M. Yan, T. Chen, Z. Liao, B. Gao, D. Su, T. Wang, S. Sharma, C. Mohan, N. Ahituv, S. Malik, Q. Z. Li, P. Su, A novel ZRS variant causes preaxial polydactyly type I by increased sonic hedgehog expression in the developing limb bud. *Genet. Med.* **22**, 189–198 (2020).

44. T. Ikeda, H. Kawaguchi, S. Kamekura, N. Ogata, Y. Mori, K. Nakamura, S. Ikegawa, U.-I. Chung, Distinct roles of Sox5, Sox6, and Sox9 in different stages of chondrogenic differentiation. *J. Bone Miner. Metab.* **23**, 337–340 (2005).

45. D. Y. Soung, Y. Dong, Y. J. Wang, M. J. Zuscik, E. M. Schwarz, R. J. O'Keefe, H. Drissi, Runx3/AML2/Cbfa3 regulates early and late chondrocyte differentiation. *J. Bone Miner. Res.* **22**, 1260–1270 (2007).

46. R. Schweitzer, J. H. Chyung, L. C. Murtaugh, A. E. Brent, V. Rosen, E. N. Olson, A. Lassar, C. J. Tabin, Analysis of the tendon cell fate using Scleraxis, a specific marker for tendons and ligaments. *Development* **128**, 3855–3866 (2001).

47. K. K. Dey, C. J. Hsiao, M. Stephens, Visualizing the structure of RNA-seq expression data using grade of membership models. *PLOS Genet.* **13**, e1006599 (2017).

48. P. Carbonetto, K. Luo, K. Dey, J. Hsiao, M. Stephens, fastTopics: Fast algorithms for fitting topic models and non-negative matrix factorizations to count data. R package version 0.4–11. (2021).

49. D. E. Wagner, C. Weinreb, Z. M. Collins, J. A. Briggs, S. G. Megason, A. M. Klein, Single-cell mapping of gene expression landscapes and lineage in the zebrafish embryo. *Science* **360**, 981–987 (2018).

50. E. Wright, M. R. Hargrave, J. Christiansen, L. Cooper, J. Kun, T. Evans, U. Gangadharan, A. Greenfield, P. Koopman, The Sry-related gene Sox9 is expressed during chondrogenesis in mouse embryos. *Nat. Genet.* **9**, 15–20 (1995).

51. B. Boehm, M. Rautschka, L. Quintana, J. Raspopovic, Ž. Jan, J. Sharpe, A landmark-free morphometric staging system for the mouse limb bud. *Development* **138**, 1227–1234 (2011).

52. G. Andrey, R. Schöpflin, I. Jerković, V. Heinrich, D. M. Ibrahim, C. Paliou, M. Hochradel, B. Timmermann, S. Haas, M. Vingron, S. Mundlos, Characterization of hundreds of regulatory landscapes in developing limbs reveals two regimes of chromatin folding. *Genome Res.* **27**, 223–233 (2017).

53. The ENCODE Project Consortium; J. E. Moore, M. J. Purcaro, H. E. Pratt, C. B. Epstein, N. Shoresh, J. Adrian, T. Kawli, C. A. Davis, A. Dobin, R. Kaul, J. Halow, E. L. Van Nostrand, P. Freese, D. U. Gorkin, Y. Shen, Y. He, M. Mackiewicz, F. Pauli-Behn, B. A. Williams, A. Mortazavi, C. A. Keller, X.-O. Zhang, S. I. Elhajjajy, J. Huey, D. E. Dickel, V. Snetkova, X. Wei, X. Wang, J. C. Rivera-Mulia, J. Rozowsky, J. Zhang, S. B. Chhetri, J. Zhang, A. Victorsen, K. P. White, A. Visel, G. W. Yeo, C. B. Burge, E. Lécuyer, D. M. Gilbert, J. Dekker, J. Rinn, E. M. Mendenhall, J. R. Ecker, M. Kellis, R. J. Klein, W. S. Noble, A. Kundaje, R. Guigó, P. J. Farnham, J. M. Cherry, R. M. Myers, B. Ren, B. R. Graveley, M. B. Gerstein, L. A. Pennacchio, M. P. Snyder, B. E. Bernstein, B. Wold, R. C. Hardison, T. R. Gingeras, J. A. Stamatoyannopoulos, Z. Weng, Expanded encyclopaedias of DNA elements in the human and mouse genomes. *Nature* **583**, 699–710 (2020).

54. I. Desanlis, Y. Kherdjemil, A. Mayran, Y. Bouklouch, C. Gentile, R. Sheth, R. Zeller, J. Drouin, M. Kmita, HOX13-dependent chromatin accessibility underlies the transition towards the digit development program. *Nat. Commun.* **11**, 2491 (2020).

55. T. Stuart, A. Butler, P. Hoffman, C. Hafemeister, E. Papalexi, W. M. Mauck, Y. Hao, M. Stoeckius, P. Smibert, R. Satija, Comprehensive integration of single-cell data. *Cell* **177**, 1888–1902.e21 (2019).

56. A. Despang, R. Schöpflin, M. Franke, S. Ali, I. Jerković, C. Paliou, W.-L. Chan, B. Timmermann, L. Wittler, M. Vingron, S. Mundlos, D. M. Ibrahim, Functional dissection of the *Sox9–Kcnj2* locus identifies nonessential and instructive roles of TAD architecture. *Nat. Genet.* **51**, 1263–1271 (2019).

57. M. Marinić, T. Aktas, S. Ruf, F. Spitz, An integrated holo-enhancer unit defines tissue and gene specificity of the Fgf8 regulatory landscape. *Dev. Cell* **24**, 530–542 (2013).

58. Z. Liu, J. Xu, J. S. Colvin, D. M. Ornitz, Coordination of chondrogenesis and osteogenesis by fibroblast growth factor 18. *Genes Dev.* **16**, 859–869 (2002).

59. S. Nassari, C. Blavet, M.-A. Bonnin, S. Stricker, D. Duprez, C. Fournier-Thibault, The chemokines CXCL12 and CXCL14 differentially regulate connective tissue markers during limb development. *Sci. Rep.* **7**, 17279 (2017).

60. G. S. Kwon, A.-K. Hadjantonakis, Eomes::GFP—A tool for live imaging cells of the trophoblast, primitive streak, and telencephalon in the mouse embryo. *Genesis* **45**, 208–217 (2007).

61. A. Zülch, M. B. Becker, P. Gruss, Expression pattern of Irx1 and Irx2 during mouse digit development. *Mech. Dev.* **106**, 159–162 (2001).

62. E. E. Storm, D. M. Kingsley, GDF5 coordinates bone and joint formation during digit development. *Dev. Biol.* **209**, 11–27 (1999).

63. R. Merino, D. Macias, Y. Gañan, J. Rodriguez-Leon, A. N. Economides, C. Rodriguez-Esteban, J. C. Izpisua-Belmonte, J. M. Hurle, Control of digit formation by activin signalling. *Development* **126**, 2161–2170 (1999).

64. J. M. Allen, E. McGlinn, A. Hill, M. L. Warman, Autopodial development is selectively impaired by misexpression of chordin-like 1 in the chick limb. *Dev. Biol.* **381**, 159–169 (2013).

65. E. Degenkolbe, J. König, J. Zimmer, M. Walther, C. Reißner, J. Nickel, F. Plöger, J. Raspopovic, J. Sharpe, K. Dathe, J. T. Hecht, S. Mundlos, S. C. Doelken, P. Seemann, A GDF5 point mutation strikes twice - Causing BDA1 and SYNS2. *PLOS Genet.* **9**, e1003846 (2013).

66. A. M. Moon, M. R. Capecchi, Fgf8 is required for outgrowth and patterning of the limbs.

Nat. Genet. **26**, 455–459 (2000).

67. P. H. Crossley, G. Minowada, C. A. MacArthur, G. R. Martin, Roles for FGF8 in the induction, initiation, and maintenance of chick limb development. *Cell* **84**, 127–136 (1996).

68. J. D. White, K. Indencleef, S. Naqvi, R. J. Eller, H. Hoskens, J. Roosenboom, M. K. Lee, J. Li, J. Mohammed, S. Richmond, E. E. Quillen, H. L. Norton, E. Feingold, T. Swigut, M. L. Marazita, H. Peeters, G. Hens, J. R. Shaffer, J. Wysocka, S. Walsh, S. M. Weinberg, M. D. Shriver, P. Claes, Insights into the genetic architecture of the human face. *Nat. Genet.* **53**, 45–53 (2021).

69. F. Kruse, J. P. Junker, A. van Oudenaarden, J. Bakkers, Tomo-seq: A method to obtain genome-wide expression data with spatial resolution. *Methods Cell Biol.* **135**, 299–307 (2016).

70. N. Karaiskos, P. Wahle, J. Alles, A. Boltengagen, S. Ayoub, C. Kipar, C. Kocks, N. Rajewsky, R. P. Zinzen, The *Drosophila* embryo at single-cell transcriptome resolution. *Science* **358**, 194–199 (2017).

71. M. Nitzan, N. Karaiskos, N. Friedman, N. Rajewsky, Gene expression cartography. *Nature* **576**, 132–137 (2019).

72. R. R. Stickels, E. Murray, P. Kumar, J. Li, J. L. Marshall, D. J. Di Bella, P. Arlotta, E. Z. Macosko, F. Chen, Highly sensitive spatial transcriptomics at near-cellular resolution with slide-seqV2. *Nat. Biotechnol.* **39**, 313–319 (2021).

73. J. V. Jun, C. M. Haney, R. J. Karpowicz, S. Giannakoulias, V. M.-Y. Lee, E. J. Petersson, D. M. Chenoweth, A “clickable” photoconvertible small fluorescent molecule as a minimalist probe for tracking individual biomolecule complexes. *J. Am. Chem. Soc.* **141**, 1893–1897 (2019).

74. A. L. Carlson, J. Fujisaki, J. Wu, J. M. Runnels, R. Turcotte, C. Lo Celso, D. T. Scadden, T. B. Strom, C. P. Lin, Tracking single cells in live animals using a photoconvertible near-infrared cell membrane label. *PLOS ONE* **8**, e69257 (2013).

75. B. Langmead, S. L. Salzberg, Fast gapped-read alignment with Bowtie 2. *Nat. Methods* **9**, 357–359 (2012).

76. M. O. Prates, C. R. B. Cabral, V. H. Lachos, Mixsmsn: Fitting finite mixture of scale mixture of skew-normal distributions. *J. Stat. Softw.* **54**, 1–20 (2013).

77. L. Albergante, E. Mirkes, J. Bac, H. Chen, A. Martin, L. Faure, E. Barillot, L. Pinello, A. Gorban, A. Zinovyev, Robust and scalable learning of complex intrinsic dataset geometry via ElPiGraph. *Entropy*. **22**, 296 (2020).

78. N. Mercader, L. Selleri, L. M. Criado, P. Pallares, C. Parras, M. L. Cleary, M. Torres, Ectopic Meis1 expression in the mouse limb bud alters P-D patterning in a Pbx1-independent manner. *Int. J. Dev. Biol.* **53**, 1483–1494 (2009).

79. S. Yokoyama, Y. Ito, H. Ueno-Kudoh, H. Shimizu, K. Uchibe, S. Albini, K. Mitsuoka, S. Miyaki, M. Kiso, A. Nagai, T. Hikata, T. Osada, N. Fukuda, S. Yamashita, D. Harada, V. Mezzano, M. Kasai, P. L. Puri, Y. Hayashizaki, H. Okado, M. Hashimoto, H. Asahara, A systems approach reveals that the myogenesis genome network is regulated by the transcriptional repressor RP58. *Dev. Cell* **17**, 836–848 (2009).

80. J. Malkmus, L. Ramos Martins, S. Jhanwar, B. Kircher, V. Palacio, R. Sheth, F. Leal, A. Duchesne, J. Lopez-Rios, K. A. Peterson, R. Reinhardt, K. Onimaru, M. J. Cohn, A. Zuniga, R. Zeller, Spatial regulation by multiple *Gremlin1* enhancers provides digit development with *cis*-regulatory robustness and evolutionary plasticity. *Nat. Commun.* **12**, 5557 (2021).

81. R. D. Riddle, M. Ensini, C. Nelson, T. Tsuchida, T. M. Jessell, C. Tabin, Induction of the LIM homeobox gene *Lmx1* by WNT6a establishes dorsoventral pattern in the vertebrate limb. *Cell* **83**, 631–640 (1995).

82. Y. Lan, P. D. Kingsley, E. S. Cho, R. Jiang, Osr2, a new mouse gene related to Drosophila odd-skipped, exhibits dynamic expression patterns during craniofacial, limb, and kidney development. *Mech. Dev.* **107**, 175–179 (2001).

83. P. Lu, Y. Yu, Y. Perdue, Z. Werb, The apical ectodermal ridge is a timer for generating distal

limb progenitors. *Development* **135**, 1395–1405 (2008).

84. J. M. Salbaum, Punc, a novel mouse gene of the immunoglobulin superfamily, is expressed predominantly in the developing nervous system. *Mech. Dev.* **71**, 201–204 (1998).
